# Structure–Property Relationship between Hard Segments of Shape Memory Polyurethane Copolymers and Interchain Hydrogen Bonds: A Comprehensive Theoretical Study

**DOI:** 10.1021/acs.jpcb.5c03305

**Published:** 2025-09-25

**Authors:** Yuliia Didovets, Mateusz Z. Brela

**Affiliations:** † Molecular Spectroscopy Group, Department of Physical Chemistry and Electrochemistry, Faculty of Chemistry, Jagiellonian University, Gronostajowa 2, PL30387 Cracow, Poland; ‡ Doctoral School of Exact and Natural Sciences, Jagiellonian University, Prof. St. Łojasiewicza St 11, PL30348 Cracow, Poland

## Abstract

Shape memory polymers have received increased interest from the scientific community, because of their extraordinary properties. Interchain hydrogen bonds play an important role in the shape memory property of polyurethane copolymers. This paper provides a comprehensive description of the interchain interactions’ network of Hard Segments within shape memory polyurethane copolymers. Model systems included Hard Segments of different chemical compositions, mainly based on hexamethylene diisocyanate (HDI), dicyclohexylmethane-4,4′-diisocyanate (HMDI), toluene-2,4-diisocyanate (TDI), and diphenylmethane-4,4′-diisocyanate (MDI). *Ab initio* molecular dynamics was used to describe the reorganization of interchain hydrogen bonds, while the character and strength of hydrogen bonds were determined with the help of interaction energy decomposition (ETS) as well as analysis of the IR spectrum data. HDI- and MDI-based Hard Segments showed the formation of interchain hydrogen bonds of the highest strength, while the TDI-based Hard Segments formed the weakest interactions. The HMDI-based Hard Segments showed medium performance. The paper also demonstrates a direct relationship between the character of interchain hydrogen bonds of the studied hard-segmented models and thermal and mechanical experimental properties of polyurethane copolymers containing corresponding fragments.

## Graphical abstract

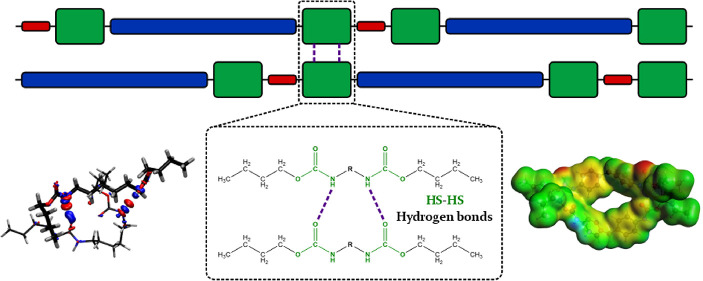

## Introduction

The variety of physicochemical properties of a polymer is strictly dependent on its chemical structure. First, the covalent structure of the polymer determines the connections between atoms within individual polymer chains. Further, the network of noncovalent interchain interactions formed between polymer chains contributes to the three-dimensional arrangement of the polymer. The type and strength of interchain interactions depend on the chemical structure of the polymer. Thus, the combination of the chemical structure specifics and the interchain interactions’ network forms a wide diversity of polymer properties, which can be observed and tested experimentally. The knowledge of the polymer’s geometry and understanding the conditions for interchain interactions’ formation in the polymer serve as an essential starting point for predicting the properties of the polymer. The ability to predict the properties of a polymer is crucial for the design of new polymer systems. The above-mentioned procedure is also used for copolymers which are more complex systems that include several chemically distinct building blocks.

The formation of interchain interactions of a particular type may require the corresponding chemical fragments present in the structure of the polymer. A good example is a hydrogen bond (HB), which can be formed only between a proton donor and an acceptor. Common donors of HBs are amine and hydroxyl groups, while carbonyl, ether, and ester groups often play the role of HBs’ acceptors. For this reason, several polymer groups, such as polyethers, polyesters, polyurethanes, and polyamides, are known for forming interchain hydrogen bonds.

Interchain hydrogen bonds are an object of frequent research in terms of polymers’ properties. Hydrogen bonds play an important role in the morphology of copolymers and polymer blends,^1–3^ mechanical properties of polymers,^4,5^ as well as shape memory^6,7^ and self-healing.^8,9^ The shape memory property is a specifically interesting example of a property that is supposed to be assisted by hydrogen bonds. Shape memory is the ability of the polymer to recover its permanent shape after deformation. The temporary shape of the polymer is created by applying mechanical force to the polymer under an external stimulus. The deformation of the polymer’s shape leads to the breaking of interchain interactions. Once the stimulus is removed and mechanical force is kept, new interchain interactions are formed, fixing the temporary shape. The temporary shape of the polymer remains stable under room conditions up to the moment when the same external stimulus is applied again. An opposite process of interchain interactions’ breaking, shape relaxation, and formation of new interchain interactions leads to permanent shape recovery.^10^

Polyurethane copolymers (PUCs) are a frequent object of research for shape memory properties. Soft and Hard Segments of PUCs provide flexible and rigid fragments to the structure of the copolymer (Figure 1). Soft Segments (SS) are usually composed of polyethers, polyesters, or polysiloxanes, while Hard Segments (HS) are built from diisocyanate moieties. Urethane bonding arises as a result of the reaction between a diisocyanate and a hydroxy-terminated Soft Segment precursor, forming a prepolymer. Prepolymer chains are then connected by chain extenders (typically diols) during the second step of the PUCs’ synthesis.^11^

**Figure 1:**
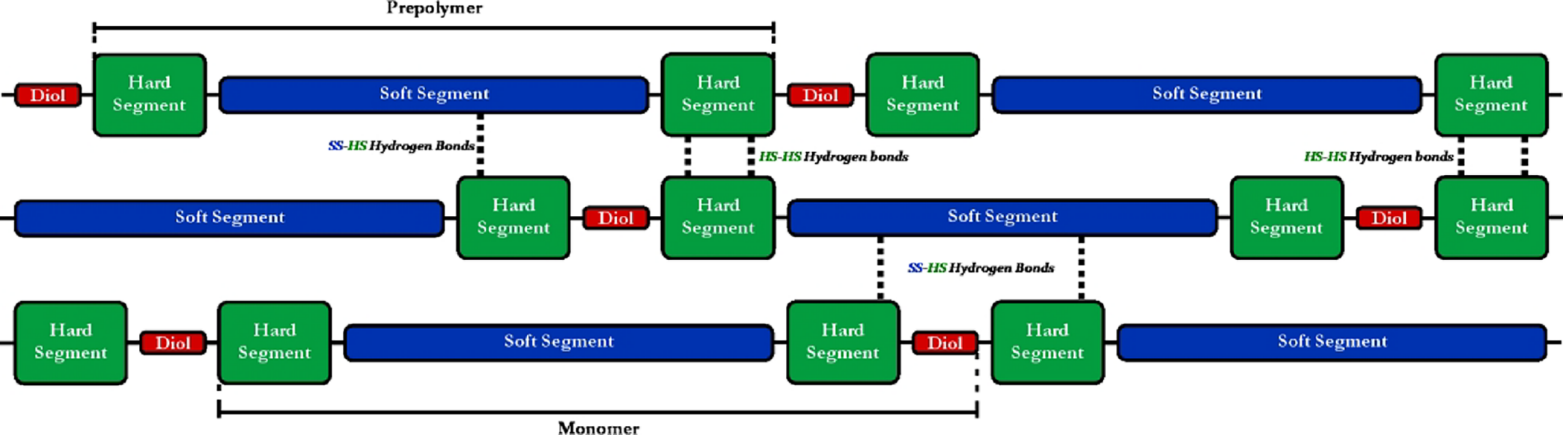
Two-segmented structure of polyurethane copolymers.

The urethane bonding, contained in PUCs, is capable of forming interchain hydrogen bonds through amine groups as donors and carbonyl groups as acceptors. Two-segmented structure, as well as interchain hydrogen bonds, make polyurethane copolymers perfect candidates for the shape memory property. Flexible Soft Segments help reorganize the copolymer’s shape, while rigid Hard Segments introduce fixing points to the structure of the copolymer, enabling shape fixation. Fixing points are created with the help of hydrogen bonds formed between urethane fragments. Further, the low energy (in comparison to covalent bonds) of HBs makes it possible to control the breaking and formation of HBs by changing the temperature of the environment. Thus, interchain HBs are believed to play a significant role in the shape-memory cycle of polyurethane copolymers.^7,12^

The structures of polyurethane copolymers contain amorphous and crystalline domains. The strength of noncovalent interactions, specifically hydrogen bonds, formed between copolymer chains contributes to the level of miscibility between Hard and Soft Segments. Weak HBs between Hard Segments and/or relatively strong HBs between Soft and Hard Segments result in better miscibility of segments, forming amorphous domains. On the other hand, strong HBs formed between Hard Segments may create large crystalline domains, leading to the microphase separation of the PUCs.^13^ Microphase separation, in turn, can assist in the shape memory properties of the PUCs.^14,15^

Interchain hydrogen bonds within PUCs are intensively studied by means of experimental measurements, including thermal tests (DSC, TGA), mechanical tests, and IR spectroscopy.^12,14–19^ The IR spectroscopy is most commonly used as the main technique for determining hydrogen bonds in the studied system. The formation of interchain hydrogen bonds leads to the decrease of the force constants of corresponding vibrations, resulting in the red shift of peaks corresponding to vibrations of groups taking part in HBs.^20^

The subject of hydrogen bonds in polyurethane copolymers was also investigated by computational chemistry approaches. Fundamental knowledge about hydrogen bonds types between urea and urethane fragments was provided mainly by Yilgör *et al.* ^21,22^ Model systems containing chemically distinct fragments making up Hard Segments in polyurethane copolymers were widely studied by means of equilibrium state calculations by Zhang *et al.*,^23–25^ by Yildirim *et al.*,^26^ and by Ren *et al.* ^27^

Deformation mechanisms and elastic properties of PUCs model systems were extensively investigated by Lempesis *et al.* ^28–31^ by means of nonequilibrium molecular dynamics simulations, as well as by Yang *et al.*,^32^ Ma *et al.*,^33^ and Afroz *et al*.^34^ using force fields. Dissipative particle dynamics was used by Yildirim *et al.* ^35–38^ to simulate the morphology of polyurethane copolymers model systems with different chemical compositions and content of Hard Segments. Other coarse-grained approaches were applied to study the shape-memory behavior of polyurethane copolymers by Uddin *et al.*,^39,40^ Abberton *et al.*,^41^ and Park *et al.* ^42,43^

All of the mentioned research papers are, without a doubt, of great scientific importance. However, regardless of valuable experimental and theoretical articles discussing interchain hydrogen bonds in shape memory polyurethane copolymers, a comprehensive description of the nature of hydrogen bonds formed between Hard Segments of different chemical compositions is still strictly necessary. This paper, therefore, focuses on providing new knowledge about the character and strength of interchain hydrogen bonds formed between Hard Segments in SMPUs. To the best of the Author’s knowledge, the dynamics of the interchain hydrogen bonds network in Hard Segments of polyurethane copolymers has never been studied by the *ab initio* approach. The aim of the current study is to apply the Born–Oppenheimer Molecular Dynamics approach to deeply investigate the reorganization of the interchain hydrogen bonds and compare the reversibility of the interchain hydrogen bonds for different models. The performed study shows direct relationships between the calculated characteristics of interchain hydrogen bonds and properties of the copolymers on a macro scale, available in the literature.

## Computational Details

Computational models for this research included two copolymer chains consisting of the chosen diisocyanate fragment and the butyl ends (Figure 2a). Butyl chains were added to the ends of the models in order to limit additional possible interchain interactions. The main parts of the models—diisocyanate-derived fragments—were chosen on the basis of available experimental data for polyurethane copolymers synthesized from hexamethylene diisocyanate (HDI), dicyclohexylmethane-4,4′-diisocyanate (HMDI), toluene-2,4-diisocyanate (TDI), and diphenylmethane-4,4′-diisocyanate (MDI) (Figure 2b). These reactants are frequently used during experimental research; therefore, spectroscopic, mechanical, and thermal properties of polyurethane copolymers’ samples are available.^12,14–19^

**Figure 2:**
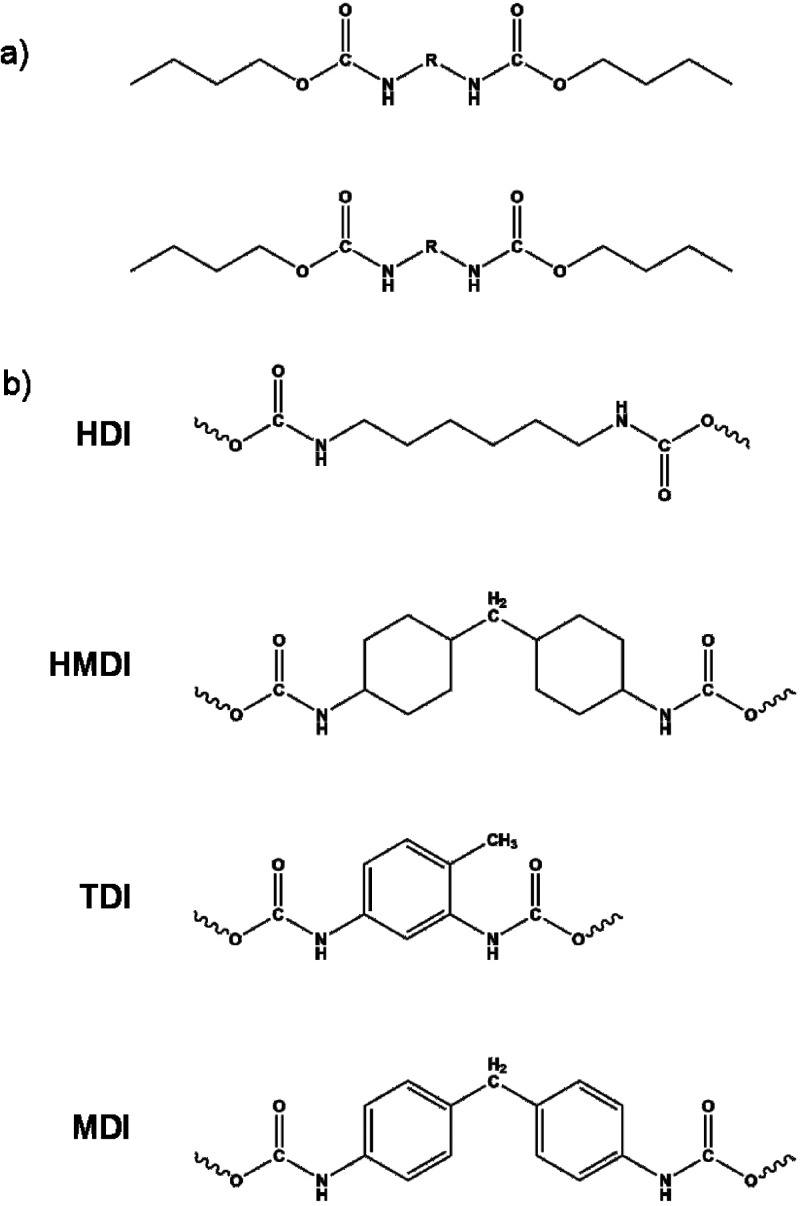
Computational models. Panel (a) shows the general structure of models, while panel (b) depicts chemical structures of the middle part of models (R).

Created computational models focus specifically on the differences between the chemical structures of the Hard-Segmented fragments. Therefore, the size of the model is limited to two chains in order to eliminate other possible interchain interactions. Computational models are capable of capturing the electrostatic, orbital, and dispersion interactions between urethane groups of copolymer chains as well as other weaker interactions. It is also possible to capture the formation of specific interchain interactions, such as hydrogen bonds, in the created models.

Ab initio molecular dynamics was performed in the CP2K software^44^ (version 2023.1) using the Quickstep scheme for all computational models analogously. Each computational model was calculated in a simulation box with a side size of 30 Å, using the canonical *NVT* ensemble. The time step was 1 fs, and the temperature was set to 300 K and controlled by the Nosé–Hoover thermostat with a constant of 1000 fs. Calculations used the BLYP/DZVP-GTH-BLYP level of theory with D3 Grimme’s dispersion correction,^45^ as well as plane waves with a cutoff equal to 450 Ry. After the first 10 ps of simulation was subtracted due to thermostatting, the total trajectories ready for analysis contained 300 ps of simulation. More details about the MD calculation procedure are provided in Section S1 of the Supporting Information.

Additionally, power spectra were obtained for all computational models as the Fourier transform of the separate atoms’ trajectories during the whole simulation.^46^ The half-width of the Gauss functions was set to 7.5 cm^–1^, and the spectrum range was from 1 to 4000 cm^–1^ with a precision of 0.5 cm^–1^.

Single-point and geometry optimization calculations were performed in SCM ADF software (version 2023.104)^47,48^ using BP86 ^49,50^ with D3 dispersion correction^45^ and the TZP basis set.

Interaction energies of systems were analyzed by the ETS energy decomposition analysis scheme.^51,52^ In this approach, the total interaction energy of the system is introduced as the interaction between fragments. The fragmentation of calculated models is depicted in Figure 3 and consists of two copolymer chains as separate fragments and the whole model as the complex.

**Figure 3:**
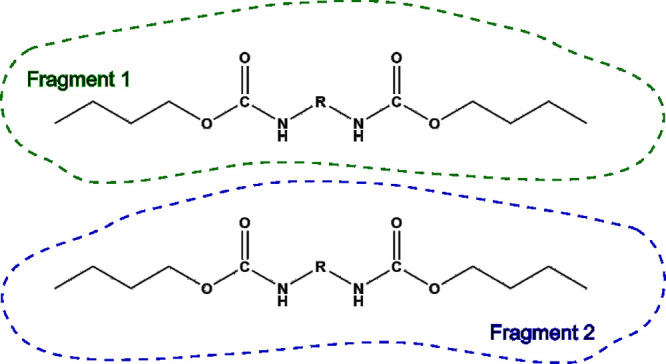
Fragmentation of computational models for ETS energy decomposition analysis.

Further, the interaction energy is decomposed into three main components: electrostatic interaction, Pauli repulsion, and orbital interaction. The first energy component relates to the Coulomb electrostatic interaction between charged fragments, and the second energy component describes the Pauli repulsion between occupied orbitals of interacting fragments, while the last energy component contributes to the interaction between occupied and virtual orbitals of each fragment and depicts the transfer of electron density within the complex. As the performed calculations additionally used the dispersion correction, the corresponding energy component was added while analyzing interaction energies of models, resulting in the equation:

$$\Delta E_{\mathrm{int}}=\Delta E_{\mathrm{elstat}}+\Delta E_{\mathrm{Pauli}}+\Delta E_{\mathrm{orb}}+E_{\mathrm{disp}}$$ 1

All interaction energy components, except the Pauli repulsion, are terms stabilizing (decreasing) the interaction energy of the system. For a deeper investigation of the interaction energies’ decomposition, an additional quantity named stabilization energy was introduced as the sum of all stabilizing components:

$$\Delta E_{\mathrm{stab}}=\Delta E_{\mathrm{elstat}}+\Delta E_{\mathrm{orb}}+E_{\mathrm{disp}}$$ 2

The contributions of the individual stabilizing components were then calculated:

$$w_{\mathrm{elstat}}=(\Delta E_{\mathrm{elstat}}/\Delta E_{\mathrm{stab}})\times 100\%$$ 3

$$w_{\mathrm{orb}}=(\Delta E_{\mathrm{orb}}/\Delta E_{\mathrm{stab}})\times 100\%$$ 4

$$w_{\mathrm{disp}}=(E_{\mathrm{disp}}/\Delta E_{\mathrm{stab}})\times 100\%$$ 5

The dominant contribution provides more information about the character of the interaction, whether it is mostly electrostatic, orbital, or dispersion.

Differential densities and molecular electrostatic maps were calculated for selected snapshots of MD simulations of each computational model using the AMSview software.^47,48^ Performing the same fragmentation of models as for all ETS calculations (see Figure 3), differential densities were calculated as the density of the complex (the whole model) decreased by the sum of the fragments’ densities. Molecular electrostatic potential maps are visualized for the entire complexes. The isovalue of the electron density for all of the chosen snapshots was set to 0.002.

ETS calculations were done for equilibrium geometries and snapshots extracted from total MD trajectories every 500 fs for each model. The NCI calculations were run using the NCIPLOT software.^53^ The visualization of computational models’ geometries was done in the VMD software.^54^

## Results and Discussion

### Interchain Interactions in the Equilibrium State

To get an introductory insight into the interchain interactions’ network as well as the relationship between structure and energy of the studied systems, the optimized geometry and corresponding electronic structure of model systems were calculated. One should, however, remember that such calculations are performed for a single geometry located at the local minimum on the potential energy surface (PES).

Geometry optimization of computational models with the applied D3 dispersion correction showed that the hard-segmented chains interact with each other (Figure 4). Differences arising due to chemical structures can be observed. Chains of the fully aliphatic HDI model interact with each other at a close distance, while the steric hindrance created by the presence of the aliphatic rings in the HMDI model results in an elongated donor–acceptor distance. For TDI and MDI models, the planar geometry of the aromatic rings decreases the steric hindrance, leading to a medium donor–acceptor distance when compared to the HDI and HMDI models.

**Figure 4:**
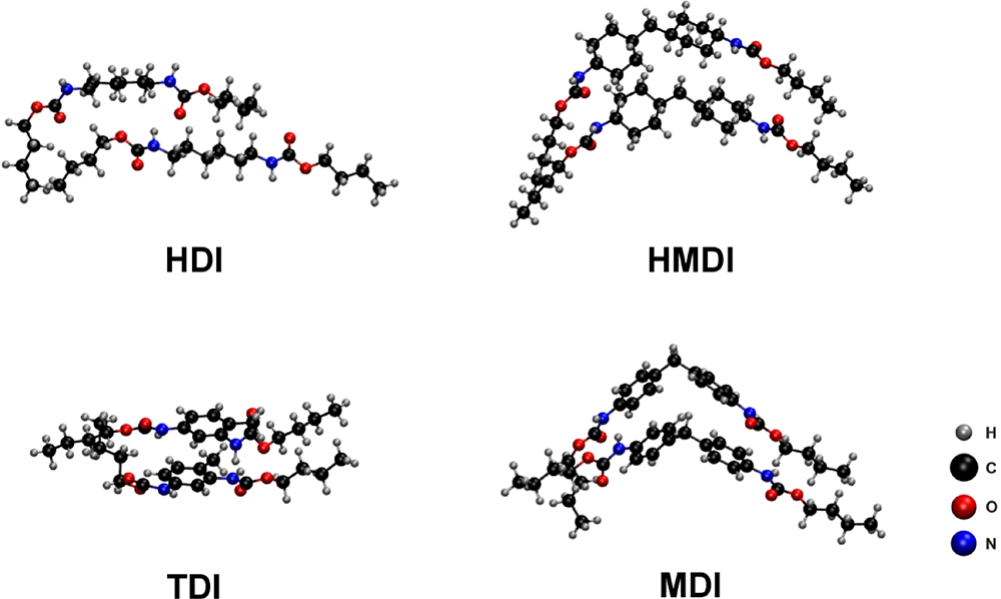
Computational models’ geometries optimized using the BP86-D3/TZP level of theory.

Energy decomposition analysis of the optimized models is presented in Table 1. The biggest interaction energy can be seen for the TDI and MDI computational models, partly due to the great components of the electrostatic interaction between copolymer chains. Models containing aromatic rings (MDI and TDI) are characterized by high delocalization of electron density. Values of the energy component corresponding to the dispersion correction are therefore high for these models, proving the significance of the dispersion interactions for the models. The biggest orbital interaction energy components can be seen for the MDI model, which suggests the biggest charge transfer between the model’s chains. Interaction energies of the HDI and HMDI models are similar but for different reasons. For the HDI model, both electrostatic and dispersion interactions have an important impact on the total interaction energy, while for the HMDI model, the biggest energy contribution is dispersion.

**Table 1:** Decomposition of Interaction Energies between Fragments of Models (Figure 3) Optimized Using the BP86-D3/TZP Level of Theory, in kcal/mol

model structure	Δ*E*_Pauli_	Δ*E*_elstat_	Δ*E*_orb_	*E*_disp_	Δ*E*_int_	Δ*E*_int_ – *E*_disp_
HDI	26.62	–15.79	–8.70	–22.23	**–20.10**	2.13
HMDI	33.67	–13.36	–9.46	–31.49	**–20.64**	10.85
TDI	39.16	–19.75	–11.14	–35.60	**–27.33**	8.27
MDI	44.72	–18.96	–12.03	–44.80	**–31.07**	13.73

The last column of Table 1 depicts the interaction energy of the models decreased by the dispersion correction. This column describes the stability of the system when the dispersion correction is not included. The results show that the dispersion correction is significant for all models, as other energy components sum up to positive values. The lowest interaction energy decreased by dispersion correction is obtained for the fully aliphatic HDI model, mainly due to electrostatic interactions. The total interaction energy hence increases in the following order: MDI > TDI > HMDI ∼ HDI.

While using equilibrium state calculations for investigations of interchain interactions, one should remember that such calculations are capable of capturing and describing the energy of the optimized system only. The geometry optimization of the system leads to the conformation corresponding to the local minimum on the PES. Global minima can be obtained more rarely from a usual geometry optimization process. Further, the geometry optimization is performed for a temperature of 0 K, which does not take thermal effects into account. Furthermore, strong interchain interactions are known to affect the vibrational spectra by shifting peaks corresponding to the changed vibration. Vibrational analysis conducted for the optimized geometry shows only the main vibrational modes, neglecting the changes in the vibrations in time.

The interchain interaction network has a dynamic nature: the interactions are constantly breaking and forming. This results in a more complex description of the interactions’ character, possible to describe with the help of molecular dynamics (MD) simulations. Molecular dynamics allows for the full conformational search of the studied system and, therefore, takes into account the reorganization of the interchain interactions’ network. The vibrations of the system are described during the MD simulations, resulting in the full spectrum containing all possible vibrations and shifts. As all structures tend to vibrate constantly at room temperature, the experimental measurements of vibrational spectra are better reproduced from the MD simulations. Analyzed MD simulations were performed in the canonical (*NVT*) ensemble, which allowed controlling the overall temperature of the system and therefore including thermal effects in the system’s vibrations.

### Dynamics of Interchain Interactions

The reorganization of the interchain interactions’ network plays a crucial role in the shape memory mechanism and is therefore significant for polyurethane copolymers investigated as candidates for the shape memory property. For all the reasons mentioned above, the molecular dynamics of all computational models was performed to obtain a clearer picture of the interchain interactions’ network.

The applied analysis procedure was analogous for all computational models. Detailed discussion of results will cover two distinct computational models: the fully aliphatic HDI model and the HMDI model containing two aliphatic rings. Such a choice for models is justified by the greatest differences in the results for these models. Results obtained for the TDI and MDI models are provided in Section S2 of the Supporting Information.

The first point in determining interchain hydrogen bonds is paying attention to geometry parameters, mainly the distances between protons and the corresponding acceptors of hydrogen bonds within the simulation. As the proton–acceptor distance typical for strong and medium HBs is from ca. 1.8 to 3.0 Å,^13^ specific attention is paid to the interactions within this distance range. Chosen distances for the HDI and HMDI models are depicted in Figures 5 and 6, respectively.

**Figure 5:**
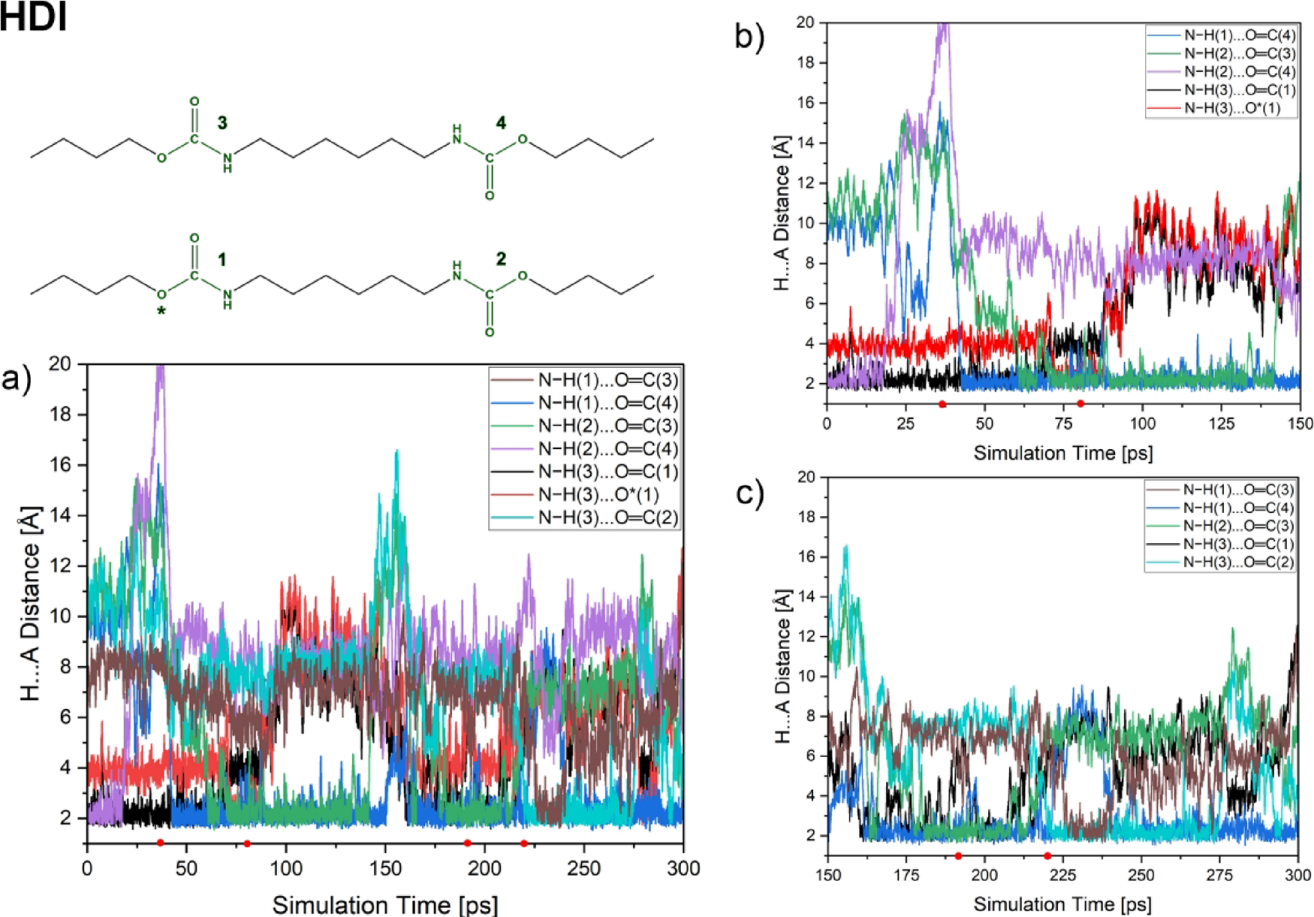
Chosen distances between protons and corresponding acceptors of hydrogen bonds during MD simulation of the HDI model. Panel a shows distances during the whole simulation of 300 ps, while panels b and c show the distances for the first and the last 150 ps of the MD simulation. Small red dots on the *X* axis depict snapshots for which the differential density and molecular electrostatic potential are demonstrated in Figure 11.

**Figure 6:**
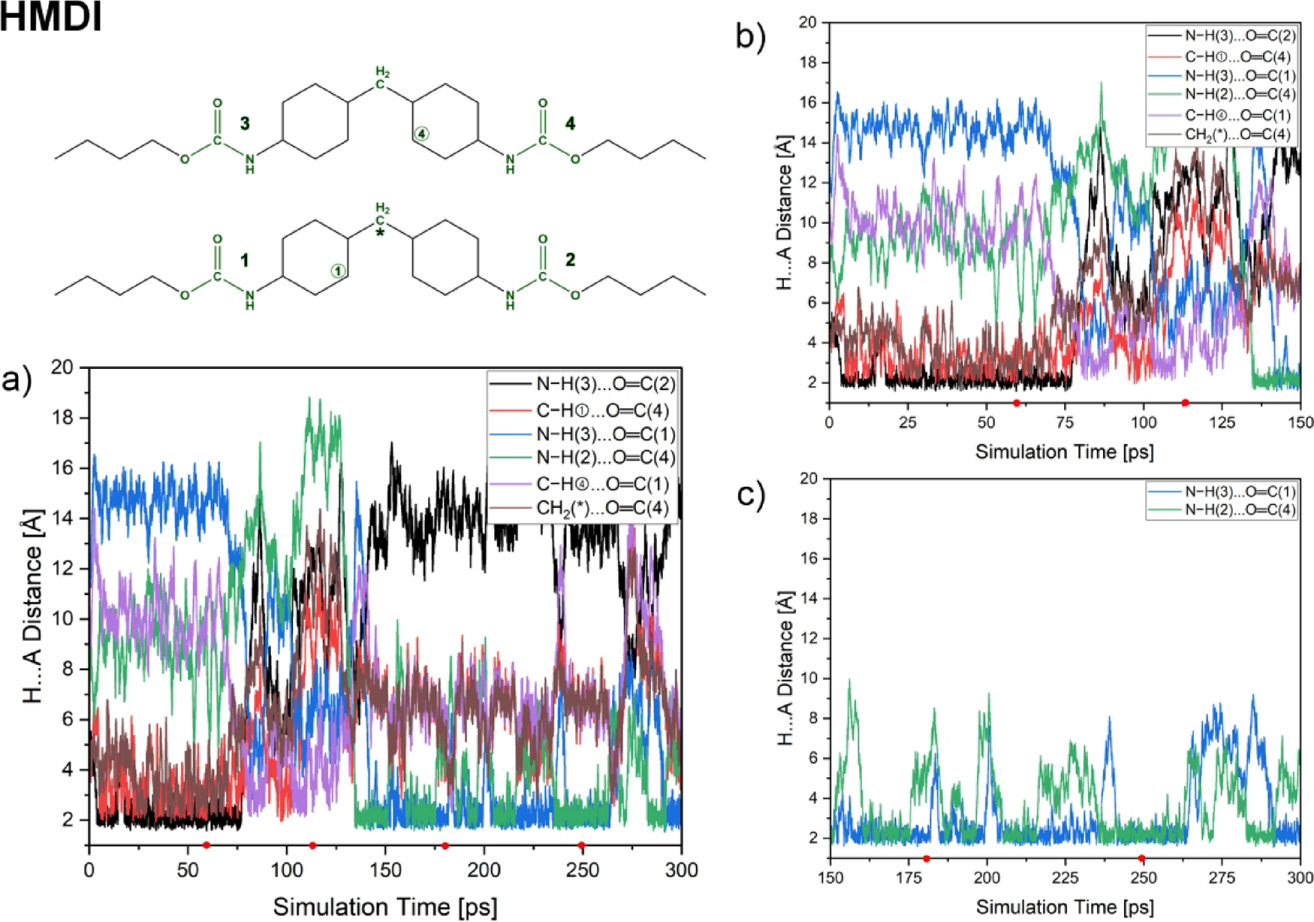
Chosen distances between protons and corresponding acceptors of hydrogen bonds during MD simulation of the HMDI model. Panel a shows distances during the whole simulation of 300 ps, while panels b and c show the distances for the first and the last 150 ps of the MD simulation. Small red dots on the *X* axis depict snapshots for which the differential density and molecular electrostatic potential are demonstrated in Figure 12.

As can be seen in Figure 5, hydrogen bonds formed within the HDI computational model are located mainly between the urethane bonding groups. During the first part (up to ca. 75 ps) of the MD simulations, the interactions were formed between the groups brought closer by constraints in the initial part of the MD simulations (groups 1 and 3, as well as groups 2 and 4; see more details in Supporting Information). The following part of the simulation shows the reorganization of the copolymer chains, placing the initially oppositely located urethane groups (1 and 4; 2 and 3) closer to the distance possible for interactions’ formation. Once formed, two main interactions, N–H(1)···O=C (4) and N–H(2)···O=C(3), remain stable until ca. 150 ps. The second half of the MD simulation shows the formation of a similar bond up to 212 ps. A new bond, N–H(1)···O=C(3), is formed for ca. 20 ps of simulation time. Finally, two bonds, N–H(1)···O=C(4) and N–H(3)···O=C(2), form so that urethane groups within the same chain participate in HBs both as donors and acceptors. The obtained MD trajectory also includes a fragment where copolymer chains are interacting only through the N–H(1)···O=C(4) channel. The breaking of the other interaction can be understood as the beginning of the following reorganization of the copolymer chains.

In the case of the HMDI computational model (Figure 6), the first part of the MD simulation (up to ca. 75 ps) is dominated by the interactions between the C=O(4) group and the methylene groups of the cyclohexane ring (1) and the methylene group located between the rings (*). A more typical N–H(3)···O=C(2), hydrogen bond is also observed. Afterward, during a significant part of the MD trajectory (up to ca. 137.5 ps), the HMDI model does not form any strong interactions, only temporary hydrogen bonds between the C=O(1) group and the methylene groups of the cyclohexane ring (4). This part of the simulation shows mainly the copolymer chains’ reorganization, taking place longer than in the case of the HDI model. Finally, new N–H (3)···O=C(1) and N–H(2)···O=C(4) hydrogen bonds are formed, suggesting the formation of the temporarily stable conformation of the copolymer chains.

Graphs demonstrating proton–acceptor distances for TDI and MDI models are depicted in Figures S2 and S3 in the Supporting Information. A large number of different hydrogen bonds are constantly formed and broken along the MD trajectories of the models. For the TDI model, which contains one aromatic ring, this is because of the additional steric hindrance between methyl substituents of the toluene fragments. In the case of the MDI model with two aromatic rings, this is mainly contributed by the bigger size of the model as well as the planar geometry of the rings.

### Spectroscopic Analysis by Power Spectra

Power spectra, obtained by the Fourier transform of the autocorrelation function of atomic positions, show the features of interchain interactions formed in the systems during the MD simulations. Combined power spectra of chosen atoms can be interpreted as spectra depicting the vibrations of the corresponding group. Such spectra, however, cannot be compared fully to the experimentally measured infrared vibrational spectra depicting the changes in the dipole moments of individual constituents of the system. In this research, power spectra obtained from atom positions are used to determine the contributions of specific atoms in the spectra.

Studied systems show formation of interchain hydrogen bonds mainly between urethane fragments, in which the amine (N–H) group usually plays the role of the hydrogen bond donor and the carbonyl group (C=O) is the acceptor of the hydrogen bond. Red shifts of peaks corresponding to amine and carbonyl groups confirm the formation of a hydrogen bond between those groups. The bigger the shift is compared to peaks of nonbonded groups, the higher the strength of the formed hydrogen bond is.^20^ As hydrogen bonds constantly form and break during MD simulations, power spectra will often show two peaks for the same group (“bonded” and “free” peaks). To determine the position of peaks corresponding to vibrations of nonbonded groups only, additional MD simulations were performed for single chains of the computational models. Power spectra are therefore used to confirm the formation of HBs and to estimate the strength order of the formed HBs.

Power spectra of the HDI and HMDI models are shown in Figure 7. The HDI model exhibited the formation and breaking of several different hydrogen bonds (Figure 5). The most bonded carbonyl group is the C=O (4), and the corresponding shift is the biggest among all groups. This carbonyl group forms HBs with two other amine groups during the simulation, mainly N–H(2)···O=C(4) and N–H(1)···O=C(4) (Figure 5). The power spectrum of the C=O(3) group shows two peaks in the C=O stretching mode, which implies that the C=O(3) group was taking part in the HB formed only during a short simulation time. Indeed, only N–H(2)···O=C(3) hydrogen bond is formed during ca. 62–137 ps, and the N–H(1)···O=C(3) HB is formed at ca. 230 ps of the simulation. The C=O(1) carbonyl group’s peak is also built up from two smaller peaks. The peak corresponding to the bonded group has a lower intensity than the one observed for the C=O (3) group. On the other hand, the peak corresponding to the ‘free’ C=O(1) carbonyl group has a bit greater intensity than for the C=O (3) group. This suggests that the C=O(1) group also formed a temporary HB during the simulation period, shorter than the C=O(3) group. One hydrogen bond, N–H(3)···O=C(1), is formed by the C=O(1) group. The remaining C=O(2) carbonyl group took part in only one, N–H(3)···O=C(2), HB along the simulation.

**Figure 7:**
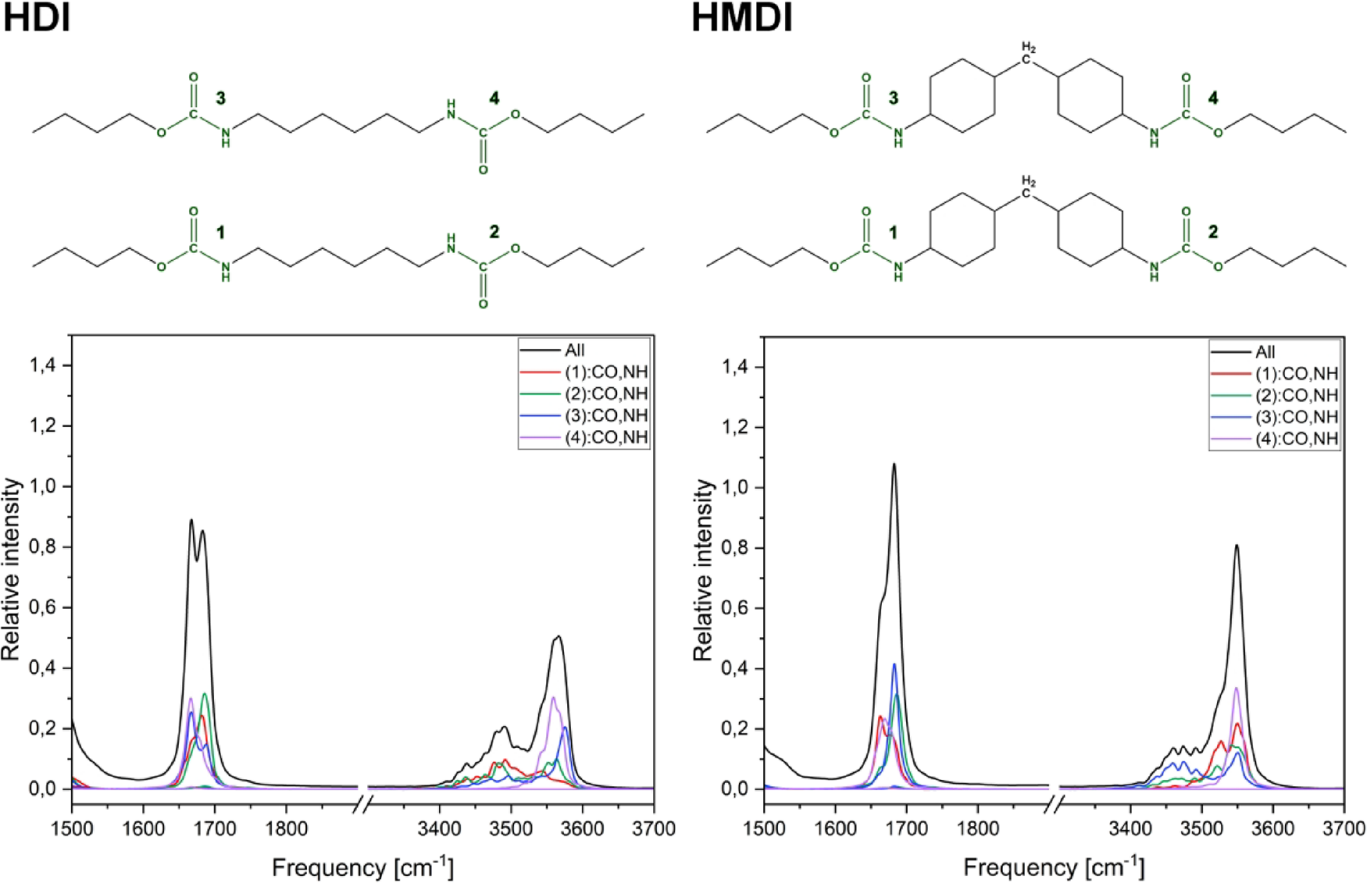
Power spectra of the HDI (left panel) and the HMDI (right panel) models for C=O and N–H stretching modes.

For amine groups, the N–H stretching region of the power spectra shows nearly the opposite order. The N–H(1) formed one strong N–H(1)···O=C(4) and one weaker N–H(1)···O=C(3) HBs during the simulation; therefore, the corresponding peak is the most shifted. It should be noted that the peak is spread from ca. 3400 to 3600 cm^–1^ because of the continuous reorganization of the first HB. The N–H(2) group formed two HBs during a shorter MD simulation time than the N–H(1) group; therefore, a small peak corresponding to the “free” N–H group is also seen on the power spectrum. The peak corresponding to the bonded N–H(3) group is also wide, with the maximum at ca. 3494 cm^–1^, due to the formation of two main hydrogen bonds, mainly N–H(3)···O=C(1) and N–H(3)···O=C(2). The N–H(4) group does not form any HBs during the whole simulation; however, there is a small shift in the corresponding peak.

The interpretation of the HMDI power spectra (right panel in Figure 7) is similar to that provided for the HDI model. Most shifted peaks in the C=O stretching mode are observed for C=O(1) and C=O(4) groups as they take part in the majority of interchain hydrogen bonds formed during the simulation (see Figure 6). Peaks corresponding to C=O(2) and C=O(3) are the least shifted, as the C=O(3) group does not form any HBs during the simulation, and the C=O(2) group forms only one hydrogen bond: N–H(3)···O=C(2). The formation of N–H(3)···O=C(2) and N–H(3)···O=C(1) hydrogen bonds also causes the red shift of the peaks for the N–H(3) group. The peak corresponding to the stretching of the N–H(2) group shows only a small shift at ca. 3525 cm^–1^, which implies a low strength of the formed N–H(2)···O=C(4) HB. The N–H (4) amine group does not take part in any HBs during the simulation; however, there is a small shift in the corresponding peak. A similar observation can be made for the peaks corresponding to the stretching vibrations of the C=O(3) and N–H(1) groups.

The formation of strong HBs by the carbonyl group (causing the red shift of the corresponding peak) often leads to the small red shift of peaks for the amine group located in the same urethane bonding and *vice versa*. This happens because the formation of a hydrogen bond results in electron density reorganization within the urethane group and, hence, higher polarization of the urethane group. This leads to the elongation of C=O and N–H bonds, decreasing the force constant of the corresponding vibrations, which can be observed as a red shift of stretching modes for amine and carbonyl groups.

Power spectra of the TDI and MDI models for C=O and N–H stretching modes are shown in Figures S4 and S5 in Section S3 of the Supporting Information.

Fragments of the power spectra corresponding to the C=O and N–H stretching modes for all computational models are shown in Figure 8. The most shifted and intensive peak corresponding to the bonded C=O group (Figure 8a) appears for the HDI model with aliphatic chains only. The lowest shift of the peak is observed for the TDI model containing one aromatic ring. Studied computational models can be sorted by the shift of C=O in order: HDI > HMDI > MDI > TDI. In the N–H stretching mode, the most separated shifted peak is shown for the HDI model. The 3400–3525 cm^–1^ region is characterized by multiple small peaks corresponding to temporarily formed HBs. The MDI and TDI models show a similar intensity of peaks in this region, whereas the shift of peaks is slightly bigger for the TDI model. The power spectrum of the HMDI model does not show a separate peak for the bonded N–H group stretching; therefore, amine groups of the model weakly take part in interchain interactions. Indeed, the HMDI model often formed hydrogen bonds of another type: between carbonyl and methylene groups.

**Figure 8:**
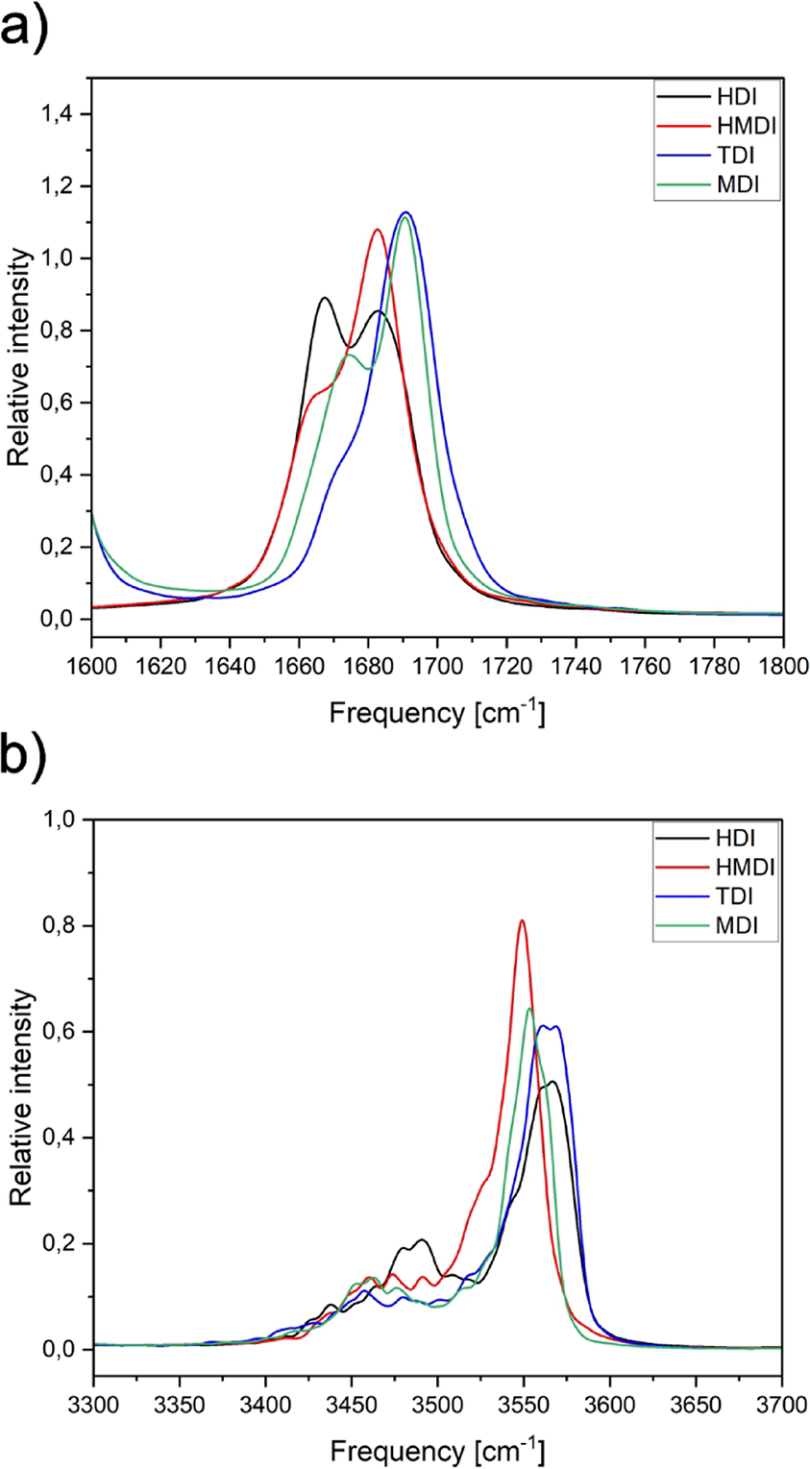
Power spectra of all computational models: (a) C=O stretching region and (b) N–H stretching region.

Calculated positions of the C=O and N–H stretching modes are gathered in Table 2. The order of peaks for the C=O stretching of noninteracting models agrees completely with the order of peaks assigned to bonded C=O groups of interacting models above. For the “free” C=O groups, the positions of corresponding peaks for HDI and HMDI models are close, resulting in mostly the same order. Apart from that, the order HDI > MDI > TDI is kept. The positions of N–H “free” stretching peaks of interacting models are in direct agreement with the corresponding positions obtained for noninteracting models.

**Table 2:** Characteristic Peaks of Carbonyl and Amine Groups for Noninteracting and Interacting Models Compared to Experimental Data, in cm^–1^ ^a^

model structure	peak	calculated for noninteracting models	calculated for interacting models	experimental^b^
HDI	ν_C=O_(bonded)		1666.5	s1684(o)^15^
				s1680(o), w1695(d)^16^
				w1662.4(o), s1687.9(d)^19^
	ν_C=O_(free)	1690.5	1685.5	m1723 ^15^
				m1720 ^16^
				m1711.2 ^19^
	ν_N–H_(bonded)		3436.5	3300 ^15^
	ν_N–H_(free)	3573	3575.5	3500 ^15^
HMDI	ν_C=O_(bonded)		1663.5	s1700(o), s1721(d)^15^
				s1699 ^17^
				w1682(o), s1696(d)^19^
	ν_C=O_(free)	1689	1686	w1738 ^15^
				m1726 ^17^
				m1720 ^19^
	ν_N–H_(bonded)		3459.5	ca.3300 ^15,17^
	ν_N–H_(free)	3557.5	3550.5	
TDI	ν_C=O_(bonded)		1673.5	from 1700 to 1730 ^12^
				w1690.1(o), s1709.2(d)^19^
	ν_C=O_(free)	1699.5	1693	s1733.1 ^19^
	ν_N–H_(bonded)		3456.5	ca.3320 ^12^
	ν_N–H_(free)	3573	3575.5	ca. 3500 ^12^
MDI	ν_C=O_(bonded)		1669	m1709(o)^15^
				m1680(o), s1700(d)^16^
				from 1700 to 1730 ^12^
				w1646.2(o), s1704.6(d)^19^
	ν_C=O_(free)	1695.5	1690.5	s1735 ^15^
				s1725 ^16^
				from 1700 to 1730 ^12^
				m1730.8 ^19^
	ν_N–H_(bonded)		3452	3300 ^15^
				ca.3320 ^12^
	ν_N–H_(free)	3563.5	3563	

a The 0.5 precision of calculated wavenumbers derives directly from the precision of the calculated spectra.

b s – strong, m – medium, w – weak, o – ordered, d – disordered.

Full power spectra for all computational models are depicted in Figure S6 in the Supporting Information.

Experimental data of the C=O and N–H stretching modes for the models are gathered in the last column of Table 2. The experimental positions^15^ of bonded C=O stretching peaks for models are as follows: HDI (1684 cm^–1^) < HMDI (1700 cm^–1^) < MDI (1709 cm^–1^) while for free carbonyl groups: HDI (1723 cm^–1^) < MDI (1735 cm^–1^) < HMDI (1738 cm^–1^). Wang et al.^19^ show a slight difference in the peak positions of ordered C=O groups: MDI (1646.2 cm^–1^) < HDI (1662.4 cm^–1^) < HMDI (1682 cm^–1^) < TDI (1690.1 cm^–1^), while peak positions for disordered and free C=O groups are similar: HDI < HMDI < MDI < TDI. The HMDI model again shows an additional coupling having a bigger shift than the MDI model for the bonded C=O group. The TDI model system shows the lowest red shifts of the corresponding stretching peaks. The order for free carbonyl groups is in line with previous analysis. Summarizing all similarities in peak shifts for the models, it is suggested that the strength of interchain HBs decreases in the order: HDI ∼ MDI > HMDI > TDI.

### Interchain Interactions Components’ Analysis

The analysis of the power spectra of models confirms the formation of interchain hydrogen bonds due to the different red shifts of peaks corresponding to stretching vibrations of carbonyl and amine groups. Peak shifts allow us to qualitatively estimate which carbonyl and amine groups take part in stronger HBs (“bonded” groups) and which remain mainly unaffected (“free” groups). A quantitative description of confirmed interchain hydrogen bonds may be provided based on a thorough analysis of interaction energies. In order to do that, interaction energies were broken down into several components with the help of the ETS energy decomposition scheme (eq 1).

Results of the ETS interaction energy decomposition analysis for the HDI and HMDI computational models are depicted in the form of graphs in Figure 9. This analysis was done for chosen snapshots of the MD simulations (Computational Details Section).

**Figure 9:**
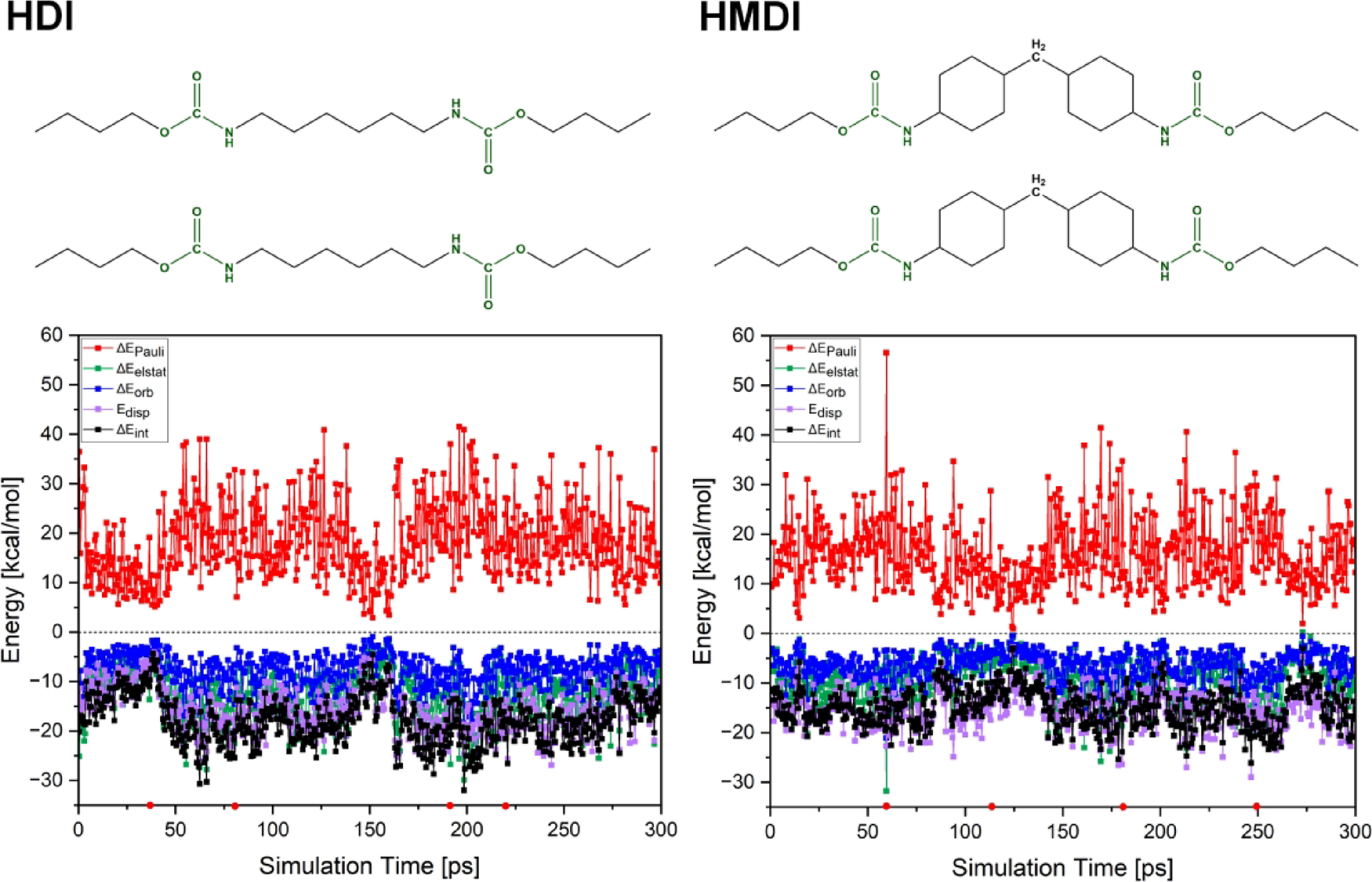
Energy decomposition analysis of snapshots from MD simulations of the HDI (left panel) and HMDI (right panel) models. Small red dots on the *X* axis depict snapshots for which the differential density and molecular electrostatic potential are demonstrated in Figures 11 and 12.

It should be noted that the positive and negative signs of the interaction energy components demonstrate the destabilizing and stabilizing characteristics of energy components. The only destabilizing component is the Pauli repulsion, while the other interaction energy components are stabilizing. It is important to remember that the comparison of different interaction energy components should be done on absolute values; therefore, the following discussion will keep up with this principle.

From the graphs in Figure 9, it can be noticed that the total interaction energy of the HDI computational model is generally lower than for the HMDI model, implying stronger interactions in the HDI model. Regions of the graphs depicting all interaction energy components being close to zero demonstrate the fragments of the MD simulations, during which the previous hydrogen bonds are broken, and the next are not formed yet; this is also usually accompanied by the models’ structures reorganization.

The analysis of the energy decomposition graphs is incomplete without comparison with the hydrogen bonds’ donor–acceptor distances depicted in Figures 5 and 6. For the HDI fully aliphatic model, interaction energy components are decreasing to zero as the first hydrogen bond

(N–H (2)···O=C (4)) is breaking. The formation of the next hydrogen bond (N–H (1)···O=C (4)) at around 37 ps leads to a significant increase in the interaction energy components. Two small peaks around 62 ps correspond to the formation of the second N–H (2)···O=C (3) hydrogen bond. The interaction energy components corresponding to the following part of the simulation do not change significantly—formed hydrogen bonds remain the same. At ca. 145 ps, the decrease of interaction energy components is seen due to the breaking of one of the hydrogen bonds. The interaction energy components increase again during the next half of the simulation due to the formation of the next HBs.

As mentioned before, interaction energy components for the HMDI model containing two aliphatic rings are lower than those observed for the HDI model. Two regions where all the components become closer to zero, at ca. 13 and 87 ps of the MD simulation, correspond to the temporary breaking of the N–H (3)···O=C (2) hydrogen bond and a complete breaking of weaker hydrogen bonds between the C=O (4) group and different methylene groups (see the top part of the Figure 6). The reorganization part of the MD simulation occurring between ca. 75 and ca. 137 ps contributed to lower values of the interaction energy components. During this part of the simulation, the dispersion correction component (violet color) is clearly seen to be higher than the total interaction energy. The increase of individual components in the final part of the simulation is caused by the formation of new N–H (3)···O=C (1) and N–H (2)···O=C (4) hydrogen bonds. It should also be noted that a single peak at about 60 ps is caused by a momentary structural reorganization. The decrease in interaction energy components seen at ca. 275 ps is explained by the following reorganization of the model.

Interaction energy components of the TDI and MDI models are shown in Figures S7 and S8 in Part S4 of the Supporting Information. Changes in the interaction energy components of these models can be analogously explained by the formation and breaking of corresponding hydrogen bonds (Figures S2 and S3).

The average values of each interaction energy component were calculated based on the Boltzmann distribution to briefly compare the results for all computational models and are demonstrated in Table 3. The average total interaction energy of the HDI model is higher than the corresponding value for the HMDI model. Apart from that, the MDI model has the biggest interaction energy, and the TDI and HMDI models’ interaction energies are comparable. If the dispersion correction is excluded, the HDI model shows the strongest stabilization, and the HMDI model shows the absence of stabilization. The order of stabilization within all models is HDI > MDI > TDI > HMDI, which is in line with the HBs strength order suggested from power spectra, and mostly similar to the one observed for the equilibrium state calculations (HDI > TDI > HMDI > MDI). It should be noted that while all models were lacking stabilization during equilibrium state calculations, molecular dynamics shows stabilization of three out of four models. The Reader is also invited to compare the individual interaction energy components from the equilibrium state and dynamic calculations, gathered in Table S1 in Section S5 of the Supporting Information.

**Table 3:** Decomposition of Interaction Energies between Fragments of Models (Figure 3)—Average Values Calculated Based on the Boltzmann Distribution of Simulation Snapshots

model structure	Δ*E*_Pauli_	Δ*E*_elstat_	Δ*E*_orb_	*E*_disp_	Δ*E*_int_	Δ*E*_int_ – *E*_disp_
HDI	17.57	–12.22	–7.36	–15.17	**–17.18**	–2.01
HMDI	16.62	–9.87	–6.51	–15.51	**–15.27**	0.24
TDI	13.85	–8.61	–5.50	–13.79	**–14.05**	–0.26
MDI	17.13	–11.12	–6.84	–17.29	**–18.12**	–0.83

Stabilization energy and its contributions were calculated for a deeper description of the interchain interactions’ character (eqs 2–5). Hydrogen bonds are formed partly due to the electrostatic interaction between a HB donor and acceptor, and partly due to the electron transfer from donor to acceptor, as well as due to the high fragments’ polarization. Therefore, when the corresponding contributions are high or at least comparable to other interactions’ contributions, one can suggest the formation of a hydrogen bond. Dispersion can be described as the electrostatic interaction between temporarily formed dipoles within the structure due to the motion of the electron density. Therefore, dispersion is common for all systems and can be higher for systems with delocalized electrons (e.g., aromatic rings). If the stabilization energy contributions are mostly equal, then the electrostatic interaction energy component is just as significant as the orbital interaction and dispersion energy. The highest contribution implies the corresponding character of the interaction.

Stabilization energy contributions for the HDI model mostly remain unchanged during the whole simulation (left panel in Figure 10). On the other hand, for the HMDI model (right panel in Figure 10), a huge increase in dispersion correction contribution is seen for the snapshots between ca. 75 and ca. 137 ps of the simulation. While the dispersion correction contribution is equal to ca. 45% of stabilization energy for the HDI model, the corresponding contribution rises to 60% for the HMDI model. This observation clearly indicates the crucial role of the dispersion correction for the stability of the HMDI model and is in line with previously introduced results. The HDI model is built up from aliphatic chains and urethane groups only, so the possibility of strong interchain interactions’ formation is high. The steric hindrance of aliphatic rings contained in the HMDI model makes it much harder for strong interactions to form between copolymer chains. Regions of the graphs in Figure 10 (e.g., at ca. 75 ps, ca. 200 ps for the HDI model and at ca. 150 ps, ca. 250 ps for the HMDI model), where the dispersion and electrostatic contributions become comparable, can be interpreted as moments of the hydrogen bonds’ formation. An increase in the orbital interaction contribution directly after this shows the charge transfer of the corresponding hydrogen bond.

**Figure 10:**
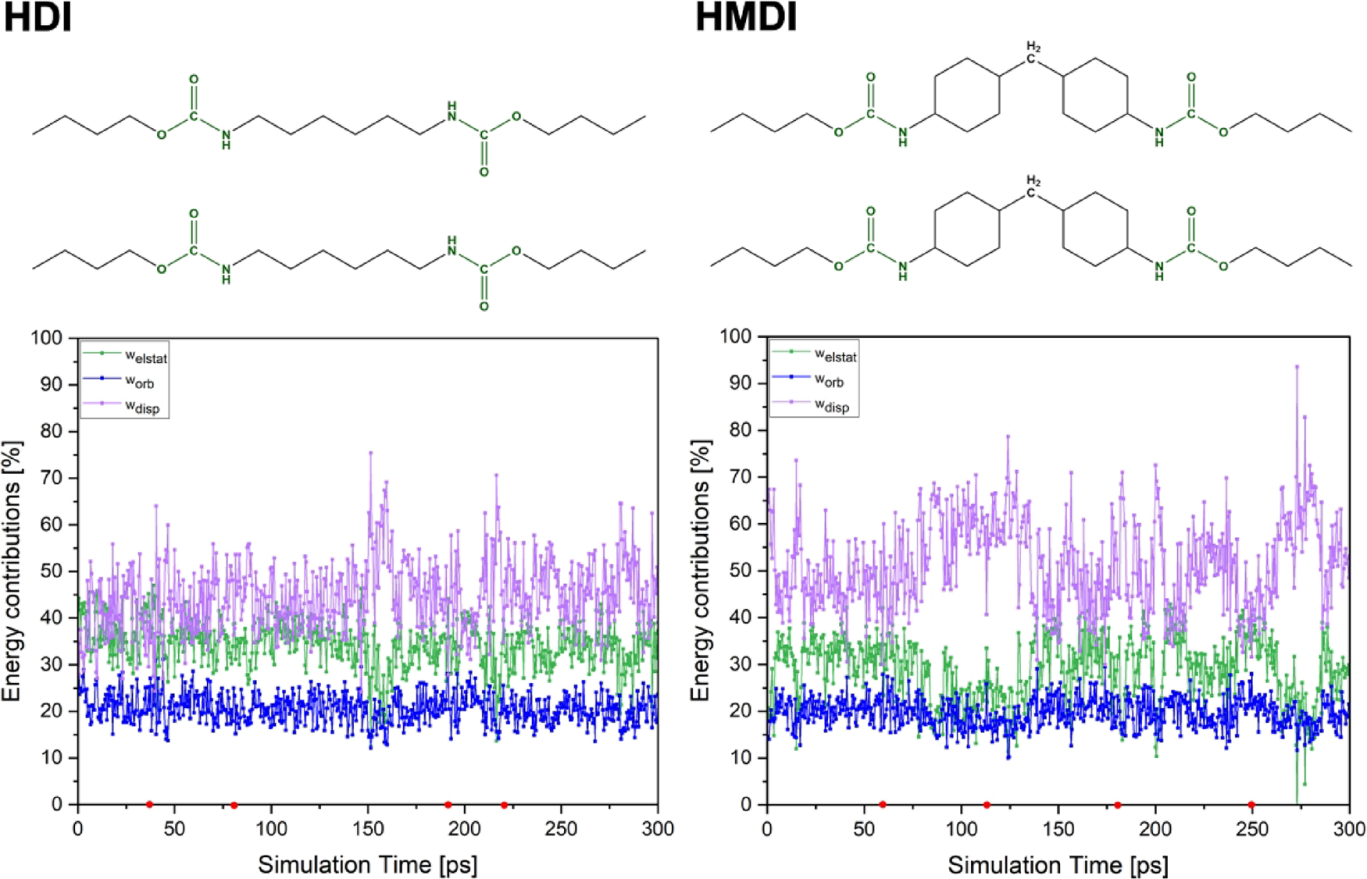
Stabilization energy contributions of snapshots from MD simulations of the HDI (left panel) and HMDI (right panel) models. Small red dots on the *X* axis depict snapshots for which the differential density and molecular electrostatic potential are demonstrated in Figures 11 and 12.

Stabilization energy contributions for the TDI and MDI models are depicted in Figures S9 and S10 in Section S6 of the Supporting Information. Stabilization energy contributions for the TDI computational model do not change significantly. The increase in the dispersion contribution, and therefore the decrease in the electrostatic and orbital contributions, is caused by the model’s conformational changes at ca. 150 ps. Dispersion correction contribution increases again when the N–H (1)···O=C (3) hydrogen bond forms, indicating a small strength of the formed interaction.

The graph of stabilization energy contributions for the MDI model shows several regions where dispersion and electrostatic interactions become comparable. This corresponds to the breaking and formation of different hydrogen bonds throughout the simulation. The dispersion correction is essential for the model’s stability during the 25–75 ps of the simulation; the highest value of dispersion contribution is 74%. This depicts the structure reorganization of the model, which happens again at ca. 137, 200, and 240 ps. This observation is particularly interesting, as it suggests the repetitiveness of the formation and breakage of interchain interactions during the simulation.

Average values of stabilizing energies as well as their contributions are gathered in Table 4 for all computational models. Additionally, Table S2 in the Supporting Information compares these values to those obtained for equilibrium state calculations. The average value of dispersion correction contribution is the highest for the TDI model, containing one aromatic ring, whereas the smallest is for the HDI model with aliphatic chains. The electrostatic interaction contribution is the highest for the HDI model, implying the highly electrostatic nature of HBs in the model. Orbital interaction contributions are similar for all models, suggesting that the main changes in the character of interchain interactions are ruled by electrostatic and dispersion interactions.

**Table 4:** Comparison of Stabilization Energy Δ*E*_stab_ and Its Components between Fragments of Models (Figure 3)—Average Values Calculated Based on the Boltzmann Distribution of Simulation Snapshots

model structure	Δ*E*_stab_ [kcal/mol]	*w*_elstat_ [%]	*w*_orb_ [%]	*w*_disp_ [%]
HDI	–34.74	34.6	20.6	44.8
HMDI	–31.90	29.7	19.8	50.5
TDI	–27.89	29.7	19.0	51.3
MDI	–35.25	31.2	19.0	49.8

The orbital and electrostatic interactions’ contributions can also be demonstrated with the help of differential densities and molecular electrostatic potential maps, respectively. Differential densities show regions where the electron density increased or decreased, depicting the electron transfer between the models’ chains. Electron density transfer from a donor to an acceptor shows the orbital interaction part of the hydrogen bond. Molecular electrostatic potential (MEP) is the measure of the electrostatic interaction between the system and the test charge at a given point in space. As electron density transfer is a significant part of HB formation, the charge distribution within the urethane groups changes, leading to a higher polarization of the groups. The change in group polarization implies a change in electrostatic interactions within the system. Therefore, the significant change in the electrostatic potential implies the formation of strong interchain hydrogen bonds. Analyzed snapshots were selected as key points of the MD simulation based on the number of possible hydrogen bonds and significant changes in interaction energy components. Selected snapshots are also marked by red dots on the *X* axes in Figures 5, 6, 9, and 10.

Differential densities and molecular electrostatic potential maps of the HDI model for chosen snapshots are depicted in Figure 11. The first snapshot corresponds to 36.5 ps of the MD simulation when one hydrogen bond, N–H (3)···O=C (1), is formed. The orbital and electrostatic interaction contributions make up ca. 25 and 45% of the stabilization energy, respectively. The differential density shows a medium electron density transfer between the C=O (1) and N–H (3) groups, confirming the presence of the charge transfer component of the formed hydrogen bonds. The formation of the N–H (3)···C=O (1) bond results in higher polarization of the corresponding urethane groups seen on the MEP. Urethane groups 2 and 4 (see top part in Figure 5) show lower polarization due to the lack of HBs’ formation.

**Figure 11:**
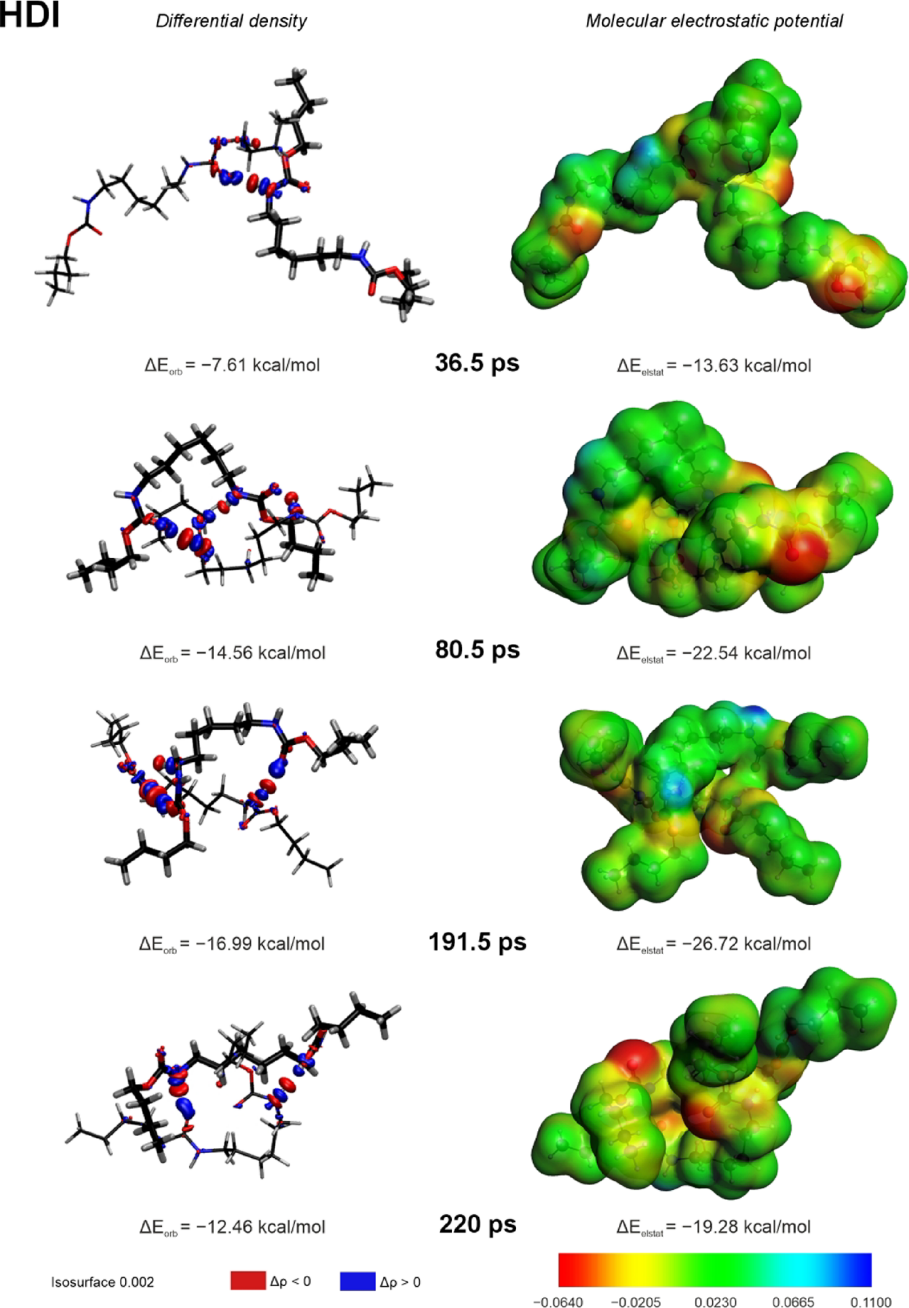
Differential densities (left panel) and molecular electrostatic potential maps (right panel) of chosen snapshots from the MD simulation of the HDI model; different thicknesses of lines are used only to distinguish individual model’s chains. The isosurface value is set to 0.002.

The second snapshot in Figure 11 corresponds to the model’s geometry at 80.5 ps of simulation, in which the same urethane group (1) takes part in the formation of two hydrogen bonds: as a donor in the newly formed N–H (1)···C=O (4) hydrogen bond and as an acceptor in the newly formed N–H (3)···O* (1) hydrogen bond. Apart from that, a third N–H (2)···C=O (3) HB is formed. The charge transfer between the mentioned groups is noticeably stronger, and the polarization of the bonded groups is significantly higher than for the previous snapshot. Increased orbital and electrostatic interaction components lead to the higher total interaction energy of the snapshot (−25.40 kcal/mol).

The 191.5 ps snapshot is characterized by the highest (among the chosen snapshots) orbital and electrostatic interaction components. The graph with hydrogen bonds’ donor–acceptor distances (Figure 5) suggests the presence of three hydrogen bonds, mainly previously formed N–H (1)···O=C (4) and N–H (2)···C=O (3), as well as newly formed N–H (3)···O=C (1) hydrogen bonds. The differential density of this snapshot confirms the charge transfer between the corresponding groups. The formed N–H (1)···O=C (4) and N–H (2)···C=O (3) hydrogen bonds induce the higher polarization of the corresponding groups, with one of the groups showing higher polarization, suggesting a higher strength of the formed interaction. Although the formation of the N–H (3)···O=C (1) hydrogen bond is not seen on the selected snapshot, the additional charge transfer between one of the carbonyl groups and one of the methylene groups is noticed.

The last 220 ps snapshot shows a bit weaker interactions by the previously formed N–H (1)···O=C (4) hydrogen bond and newly formed N–H (3)···O=C (2) hydrogen bond. The corresponding orbital and electrostatic interaction components are lower than those for the previous two snapshots. The formation of HBs within this snapshot also leads to a change in the polarization of methylene fragments around the urethane groups.

Differential densities and MEPs for the selected snapshots of the HMDI model are depicted in Figure 12. For the first snapshot, corresponding to 59.5 ps of the MD simulation time, the formation of three weak hydrogen bonds can be observed: between the C=O (4) group and one of the C–H groups of the first aliphatic ring, between the C=O (4) group and the methylene group, and between the N–H (3) and the C=O (2) groups (see Figure 6). The first snapshot is also characterized by the highest electrostatic interaction component within all HMDI snapshots due to numerous hydrogen bonds formed in the model. The polarization of urethane group 3 (on the right of the model) is significantly higher than the polarization of group 1 (on the left of the model) because of the formation of the N–H (3)···C=O (2) hydrogen bond.

**Figure 12:**
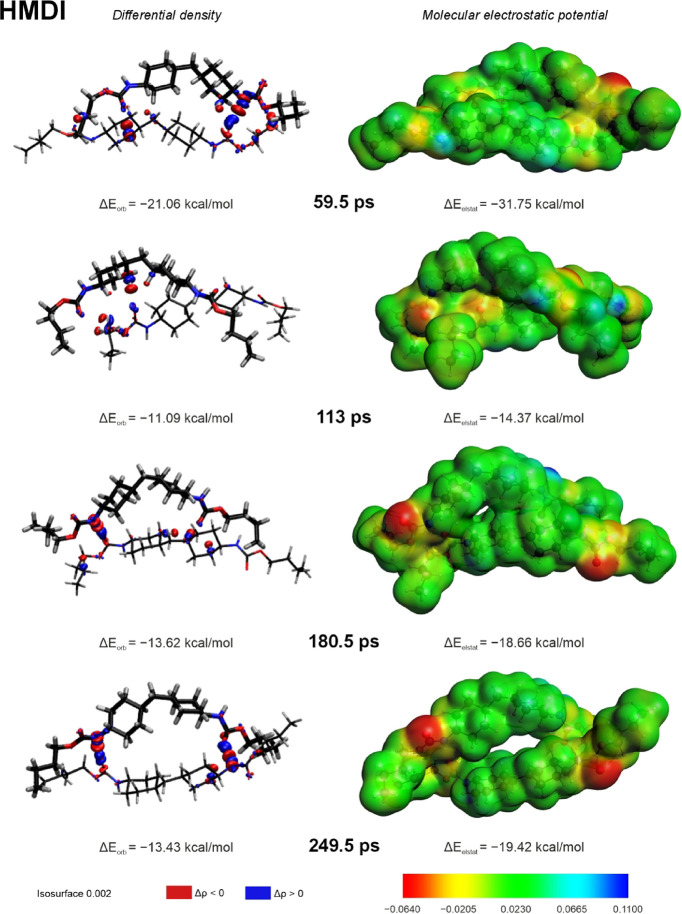
Differential densities (left panel) and molecular electrostatic potential maps (right panel) of chosen snapshots from the MD simulation of the HMDI model; different thicknesses of lines are used only to distinguish individual model’s chains. The isosurface value is set to 0.002.

The next snapshot (113 ps) is characterized primarily by one newly formed hydrogen bond between the C=O (1) group and one of the C–H groups of the fourth aliphatic ring. A slight charge transfer is also seen between the C=O (4) group and one of the C–H groups of the butyl chain. The polarization of the model is the lowest among all of the analyzed snapshots, as the HMDI model does not form interchain HBs in this snapshot. Therefore, the corresponding electrostatic interaction component describes mainly nonspecific interactions within the model.

During the two following 180.5 and 249.5 ps snapshots, two hydrogen bonds, N–H (3)···O=C (1) and N–H (2)···O=C (4), are formed. The polarization of the urethane group 2 (on the right of the model) is lower in the 180.5 ps snapshot than in the 249.5 ps snapshot, as the second N–H (2)··· O=C (4) hydrogen bond forms only in the 249.5 ps snapshot. Similar values of orbital and electrostatic interactions’ components can be noticed for the snapshots. The increase of the electrostatic interaction contributions (from ca. 32 to ca. 40%) can be explained by the formation of the N–H (2)··· O=C (4) hydrogen bond for the final snapshot at 249.5 ps.

Similar analysis of the orbital and electrostatic interactions of the TDI and MDI models can be performed based on differential densities and molecular electrostatic potential maps of the chosen snapshots depicted in Figures S11 and S12 in Section S7 of the Supporting Information.

In order to additionally confirm the formation of hydrogen bonds between copolymer chains of the models, NCI calculations were run for selected snapshots of the models. Noncovalent interactions are established based on the calculation of the reduced density gradient. The character of the interaction is determined based on the sign of the second eigenvalue of the Hessian, λ_2_: high negative values of the sign­(λ_2_)­ρ correspond to attractive interactions (such as hydrogen bonding), high positive values correspond to nonbonding interactions, and values close to zero correspond to van der Waals interactions.^55,56^

The reduced density gradient graphs of the snapshots are presented in Figures S13–S16 in Section S8 of the Supporting Information. The selected noncovalent interactions of the models’ snapshots are depicted in Figures S17–S20 in the Supporting Information. All previously suggested hydrogen bonds are clearly seen in the NCI plots, with the different colors depicting different strengths of individual interactions.

### Interchain Interactions vs Experimental Properties

The comparison of the computationally obtained results with the experimentally determined properties of the corresponding polyurethane copolymers is an important part of this study. This section of the paper aims to determine the main dependences between the nature of interchain noncovalent interactions in Hard Segments of PUCs simulated by means of computational chemistry methods and experimentally measured physicochemical properties of the synthesized polyurethane copolymers reported in the literature. As the created model systems contained hard-segmented fragments only, the results of this comparison should be useful for determining which property, and to what extent, is mainly controlled by interchain interactions in Hard Segments of PUCs.

Glass transition or melting temperatures, as well as tensile stress at 200% strain of PUCs samples containing the studied Hard Segments, are presented in Table 5. The gathered data were mostly taken from published experimental articles.^14,15,19^ The stress/strain ratio was calculated for the maximum values of tensile stress and strain provided for the corresponding copolymer sample. As the synthesis and experimental measurements are performed analogously for samples within articles,^14,15,19^ the quantitative comparison of Hard Segments should be done on the results for every article separately. However, a qualitative comparison of Hard Segments in terms of phase transition temperatures and mechanical properties is provided to suggest a general structure–property relationship.

**Table 5:** Selected Thermal and Mechanical Properties of PUCs Containing the Studied HS

hard segment	glass transition *T*_g_^‡^/melting temperature, *T*_m_^†^ [°C]	tensile stress at 200% strain [MPa]	stress/strain ratio [MPa]^a^	reference
HDI	–69.4^‡^		7.50	^19^
HMDI	–30.2^‡^	11.25	5.17	
TDI	–17.1^‡^	1.25	0.41	
MDI	–41.8^‡^	11.25	4.07	
HDI	46.6^†^; 49.0^†^	7.9	3.95	^14^
TDI	48.9^†^; 52.9^†^	3.7	1.85	
MDI	48.6^†^; 54.1^†^	5.6	2.80	
HDI	–78.0^‡^		14.00	^15^
HMDI	–78.0^‡^		2.32	
MDI	–71.0^‡^		1.05	

a Calculated based on cited experimental data for values on break, if available.

Glass transition and melting temperatures of PUCs containing the studied HSs are compared to average values of HBs formed between chains during the MD simulation of the corresponding model, presented in Table S3. The average interaction energy per HB in the models is as follows: HMDI < MDI < TDI < HDI, meaning that the MDI model forms the strongest interchain HBs, while the HDI model forms the weakest HBs. The order of glass transition temperatures of copolymers containing the studied HSs^19^ is as follows: HDI < MDI < HMDI < TDI. This sequence is mainly in line with the previously stated average interaction energy per HB in the computed models. The TDI-Hard Segmented copolymer is an exception, showing nearly the lowest calculated average interaction energy per HB and simultaneously having the highest glass transition temperature in the experiments.^19^ Similar observation can also be seen from the first measurement of the melting temperatures in ref 14. This implies that the TDI Hard Segment may have other contributions to its stability, for example, additional conformation change limitations due to the methyl substituent. When comparing melting temperatures of PUCs samples from ref 14, the second measurement (the second value), one can notice a complete agreement of the order of HSs: HDI< TDI < MDI. The highest melting temperature of the PUC sample containing MDI moieties correlates with the highest average strength of the single HB in the MDI model. Further, the highest value of glass transition temperature of the MDI-containing PUC is observed as the result of another experiment.^15^ The HDI- and HMDI-containing PUCs showed similar glass transition temperatures.^15^ For these models, the balance between the number and average strength of HBs in the models could be the reason.

The amount of tensile stress applied to the copolymer sample to obtain a 200% strain was chosen as a way of aligning results to the same level of strain. The focus is on the amount of force needed for the copolymer samples of different chemical compositions of Hard Segments. For ref 14, the amount of force decreases in order: HDI > MDI > TDI, implying the fully aliphatic HDI model to be the most mechanically resistant one. The interaction energy for the HDI model obtained from MD calculations is only 1 kcal/mol higher than for the MDI model, thus suggesting similar properties of HDI and MDI models. The preference for the HDI Hard Segment is also shown as the highest value of tensile stress–strain ratio in refs 15 and 19 . Different order, as well as similar interaction energies of HDI and MDI models, suggest that these models have a similar behavior in PUCs. Additionally, PUCs samples containing HDI and MDI moieties were proven to increase the microphase separation of PUCs, as crystallization peaks were observed during thermal tests of the samples.^14,15^ Further, while the average strength of the HB in these models is different, the HDI model forms two HBs simultaneously more often than the MDI model (Table S3). The MDI Hard Segment shows higher tensile stress than the TDI Hard Segment in refs 14 and 19 , which agrees with the interaction energy of the MDI model being 4 kcal/mol lower than the corresponding energy of the TDI model. Comparing the tensile stress–strain ratios of HDI and HMDI Hard Segments in refs 15 and 19 , it can be seen that the HMDI Hard Segment has weaker mechanical properties than the HDI Hard Segment. This is in line with the ca. 2 kcal/mol higher interaction energy for the HMDI model compared to the HDI model. The PUCs samples containing TDI Hard Segment showed significantly lower mechanical properties than other HSs, due to the formation of temporary HBs, only one during most of the MD simulation of the model. Therefore, it is suggested that the computationally derived order of interaction energies of HS models: MDI < HDI < HMDI < TDI, mostly agrees with the experimental results for PUCs composed of these HSs.

## Conclusions

The comprehensive study focused on interchain hydrogen bonds between Hard Segments of polyurethane copolymers with different compositions of HSs. Model systems included diisocyanate fragments based on hexamethylene diisocyanate (HDI), dicyclohexylmethane-4,4′-diisocyanate (HMDI), toluene-2,4-diisocyanate (TDI), and diphenylmethane-4,4′-diisocyanate (MDI). The applied computational procedure was successful in capturing the dynamic nature of interchain interactions in Hard Segments of polyurethane copolymers. While ab initio molecular dynamics allowed describing the constant reorganization of the interchain interactions’ network, equilibrium state calculations and additional analysis (power spectra, ETS energy decomposition analysis, differential densities, and molecular electrostatic potential maps) resulted in a comprehensive description of the interchain interactions’ strength and character. Further, the obtained results are easy to compare with selected thermal and mechanical experimental data. Therefore, the performed computational protocol can certainly be used for the research of polyurethane copolymers’ Hard Segments of different chemical structures.

The MDI hard segment, composed of two aromatic rings, was identified to form the strongest interchain HBs. The fully aliphatic HDI model was established to show similar properties, with a balance of slightly lower strengths but a greater amount of HBs. The HMDI model, containing two aliphatic rings, formed medium-strong HBs in a smaller amount, additionally stabilized by dispersion interactions. The TDI model with one aromatic ring showed the poorest overall performance, despite the second highest strength of a single HB formed in the model, due to constant breaking and formation of single hydrogen bonds within the MD simulation.

Computationally obtained results were also compared to available thermal and mechanical experiments.^14,15,19^ The similarities in mechanical and thermal properties of the HDI- and MDI-containing PUCs (Table 5) are confirmed by similar peak shifts in power spectra (Table 2) and high average interaction energies (Table 3) for models containing corresponding diisocyanate moieties. High polarization of the corresponding models (Figures 11 and S12) additionally underlines the significance of electrostatic interactions in interchain interactions of the models. The experimental properties of the PUCs with toluene-based Hard Segments are directly related to the less stable interchain hydrogen bonds (Figure S2), low peak shifts on power spectra (Figure S4), and the lowest interaction energy of the TDI-based computational model within all studied systems. The “medium” performance of the HMDI-based PUCs is explained through a combination of different peak shifts on power spectra for carbonyl and amine stretching regions (Figure 7, right panel) and the medium interaction energy (Table 3), in particular, high orbital interaction (Figure 12, left panel) and the lowest polarization (Figure 12, right panel). The determined order of interchain interactions’ strength is therefore as follows: MDI ∼ HDI > HMDI > TDI. The established structure–property relationship of polyurethane copolymers’ Hard Segments will undoubtedly be useful for the following research on shape memory and microphase separation in smart polymers.

## Supplementary Material











## Notes

Computational procedure, geometry parameters for the TDI and MDI models, power spectra for other models, ETS energy decomposition analysis for the TDI and MDI models, calculation of average values based on the Boltzmann distribution, stabilization energy contributions for the TDI and MDI models, differential densities and molecular electrostatic maps for the TDI and MDI models, noncovalent interactions calculations, and number and strength of HBs during MD simulations (PDF)
MD simulation of the HDI model (MP4)
MD simulation of the HMDI model (MP4)
MD simulation of the TDI model (MP4)
MD simulation of the MDI model (MP4)

## ABBREVIATIONS

PUC: polyurethane copolymer
HS: Hard Segment
SS: Soft Segment
HB: hydrogen bond
MD: molecular dynamics
PES: potential energy surface
ETS: extended transition state
HDI: hexamethylene diisocyanate
HMDI: dicyclohexylmethane-4,4′-diisocyanate
TDI: toluene-2,4-diisocyanate
MDI: diphenylmethane-4,4′-diisocyanate
IR: infrared spectroscopy
DCS: differential scanning calorimetry
TGA: thermogravimetric analysis
