# Supplementary material for: Structure–Property Relationship between Hard Segments of Shape Memory Polyurethane Copolymers and Interchain Hydrogen Bonds: A Comprehensive Theoretical Study
Source: J Phys Chem B. 2025 Sep 25;129(40):10504–20. doi: 10.1021/acs.jpcb.5c03305 (PMC12516715; doi:10.1021/acs.jpcb.5c03305)
Supplement: Supplementary file 1 [file jp5c03305_si_001.pdf]

Structure-property relationship between Hard Segments of Shape Memory Polyurethane Copolymers and Interchain Hydrogen Bonds: a Comprehensive Theoretical Study

Supporting Information

Yuliia Didovets†‡, Mateusz Z. Brela†.*

†Molecular Spectroscopy Group, Department of Physical Chemistry and Electrochemistry, Faculty of Chemistry, Jagiellonian University, Gronostajowa 2, PL30387 Cracow, Poland.

‡ Doctoral School of Exact and Natural Sciences, Jagiellonian University, Prof. St. Łojasiewicza St 11, PL30348, Cracow, Poland.

Contents:

S1. Computational procedure
S2. Geometry parameters for the TDI and the MDI model
S3. Power spectra for other models
S4. ETS Energy Decomposition Analysis for the TDI and the MDI model
S5. Calculation of average values based on the Boltzmann distribution
S6. Stabilization energy contributions for the TDI and the MDI model
S7. Differential densities and molecular electrostatic maps for the TDI and the MDI model
S8. Noncovalent Interactions calculations
S9. Number and strength of HBs during MD simulations

S1. Computational procedure

First, to bring copolymer chains closer, MD simulations were performed with constraints for distances between neighbouring urethane groups (**Figure S1**). The constraints' growth was −0.00025Å/fs, while the target distance of constraints was set to ca. 2.15 Å. The timestep was 1 fs, the temperature was set to 300 K and controlled by a Nosé-Hoover thermostat with a constant of 1000 fs. Calculations used BLYP/DZVP-GTH-BLYP level of theory with D3 Grimme's dispersion correction, as well as plane waves with a cutoff equal to 450 Ry. Each computational model was calculated in simulation boxes with a side size of 30 Å using the canonical NVT ensemble.

After reaching the target value, constraints were disabled, and molecular dynamics calculations were then performed. The first 10 ps of the obtained trajectory was cut out from the analysis in order to investigate the 300 ps of thermally stabilized simulation.

Single copolymer chains of models depicted in **Figure 2** were also simulated by ab initio molecular dynamics. The computational procedure was analogous to the free dynamics performed for two-chained models (**Figure 2**).

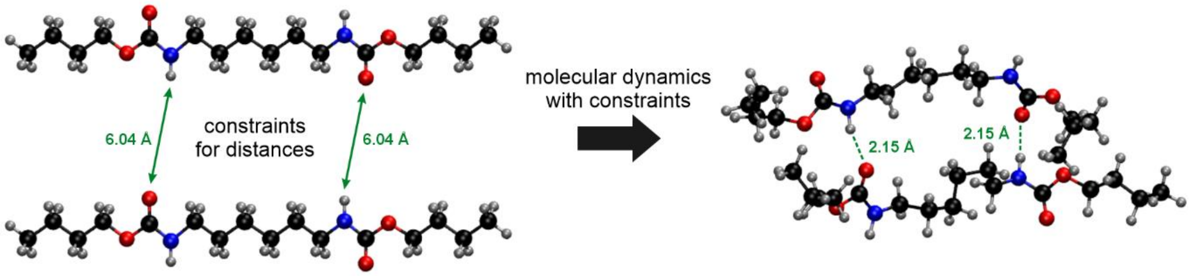

Figure S1. Constraints used in the initial part of the molecular dynamics, on the example of the HDI model.

S2. Geometry parameters for the TDI and the MDI model

For the TDI model, containing one aromatic ring, one or two hydrogen bonds are mostly formed. The first part of the simulation shows the dominance of two hydrogen bonds, N–H(1)…O=C(4) and N–H(3)…O=C(1), in which the first urethane group plays the role of both hydrogen bond donor and acceptor, respectively. The hydrogen bond between urethane groups 2 and 4 is not, however, seen, because of the increase in steric hindrance between the methyl substituents of toluene fragments. From ca. 125 ps to ca. 140 ps of the simulation, the structure reorganization led to a decrease in the number and stability of hydrogen bonds. The second half of the simulation shows breaking and formation of different bonds, mainly N–H(1)…O=C(4), N–H(1)…O*(3) and N–H(3)…O=C(2).

The MDI model with two aromatic rings shows two hydrogen bonds at the beginning of the simulation (up to ca. 37 ps). After the reorganization of the model's chains, two main hydrogen bonds are created: N–H(3)…O=C(2) and N–H(1)…O=C(4). These HBs are formed between the opposite sides of the model, implying the necessity of rotation of the model's chain in order to form a conformationally favourable geometry. After the second reorganization of the model at ca. 160 ps, two main bonds are formed, mainly N–H(2)…O=C(4) and N–H(3)…O=C(1).

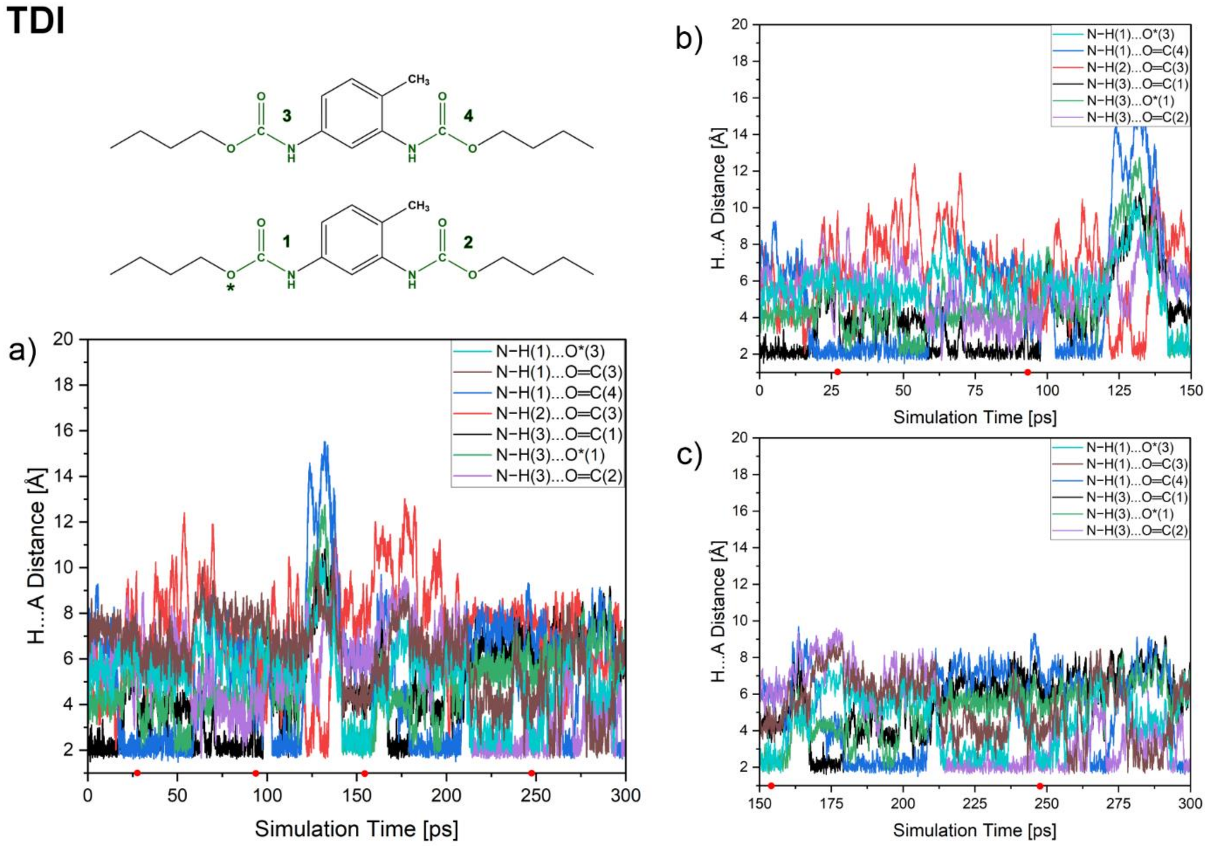

Figure S2. Chosen distances between protons and corresponding acceptors of hydrogen bonds during MD simulation of the TDI model. Panel a shows distances during the whole simulation of 300ps, while panels b and c show the distances for the first and the last 150ps of the MD simulation. Small red dots on the X axis depict snapshots for which the differential density and molecular electrostatic potential are demonstrated in **Figure S11**.

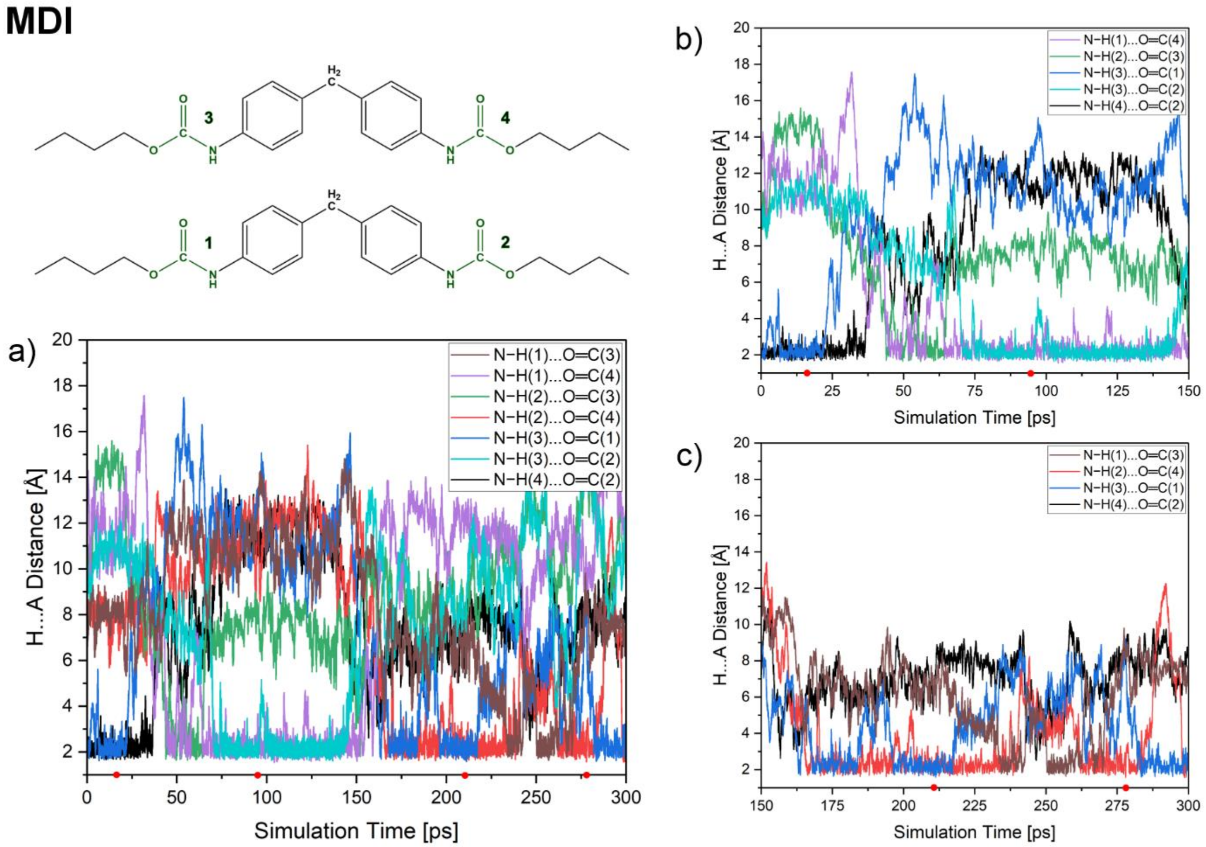

Figure S3. Chosen distances between protons and corresponding acceptors of hydrogen bonds during MD simulation of the MDI model. Panel a shows distances during the whole simulation of 300ps, while panels b and c show the distances for the first and the last 150ps of the MD simulation. Small red dots on the X axis depict snapshots for which the differential density and molecular electrostatic potential are demonstrated in **Figure S12**.

S3. Power spectra for other models

Computational models discussed in the manuscript include two Hard-Segmented chains, therefore describing the interchain interactions (interacting models). For the purpose of comparison, similar models containing only one Hard-Segmented chain were calculated to obtain the power spectra peaks characteristic for C=O and N–H groups of the models (non-interacting models).

The differences between the wavenumbers of peaks between calculated models and experimental results may be derived from the partial composition of computational models, containing HS only, as well as the size of the studied system. However, the differences are ca. 20-30 cm^{-1}, rarely 40 cm^{-1} for C=O stretching mode, and 130-150 cm^{-1} for N–H stretching modes. The comparison between calculated and experimental peaks is therefore not quantitative, but qualitative, focusing mainly on general relationships between the chemical composition of the model and peak shifts. The presented data for bonded C=O and N-H groups are shown as the most shifted peak observed for one or several groups (see **Figures 7** and **S4-S5**). Similarly, peaks assigned to 'free' C=O and N-H groups are the ones least shifted.

Stretching vibration peaks calculated for non-interacting one-chain models show the 'default' peak positions derived from intramolecular interactions and couplings. For non-interacting models, the order from the minimum to maximum wavenumber for C=O stretch is as follows: HMDI (1689 cm^{-1}) < HDI (1690.5 cm^{-1}) < MDI (1695.5 cm^{-1}) < TDI (1699.5 cm^{-1}). This order suggests the biggest amount of intramolecular interactions for the HMDI model, containing two aliphatic rings. The MDI model, containing two aromatic rings, shows possible couplings with

aromatic rings. The TDI model with one aromatic ring has a similar nature to the MDI model, but with a lesser number of aromatic rings.

For the N-H stretching peaks, the order of models is similar except for the replacement of MDI and HDI models: HMDI (3557.5 cm^{-1})<MDI (3563.5 cm^{-1})<HDI (3573 cm^{-1}) = TDI (3573 cm^{-1}). The N-H groups are directly connected to aromatic rings of the MDI model, therefore, the coupling could be bigger for this group and smaller for the following carbonyl C=O group.

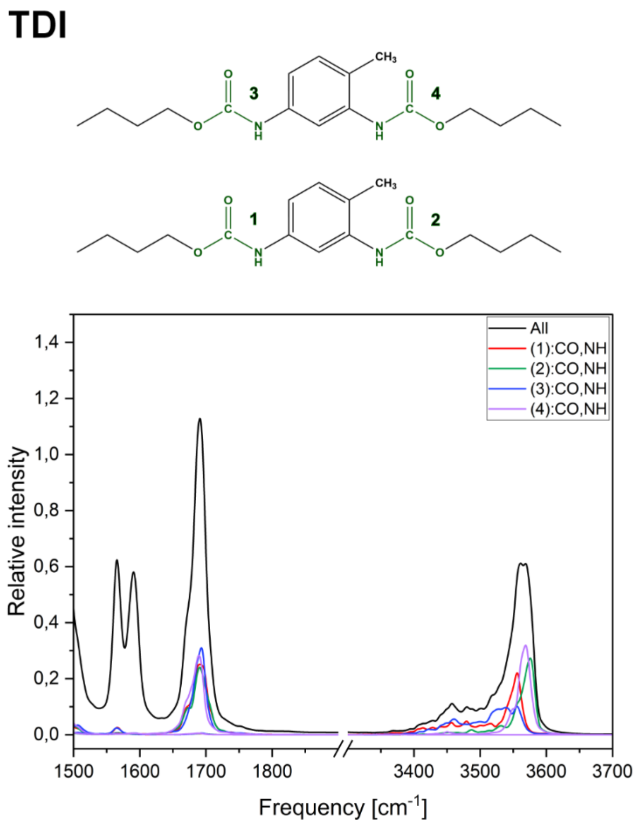

Figure S4. Power spectra of the TDI model for C=O and N-H stretching modes.

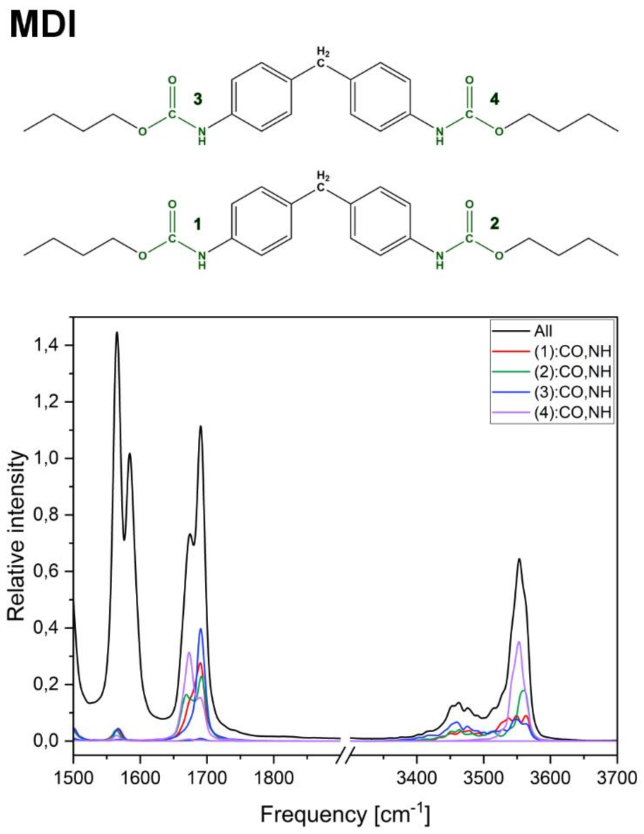

Figure S5. Power spectra of the MDI model for C=O and N-H stretching modes.

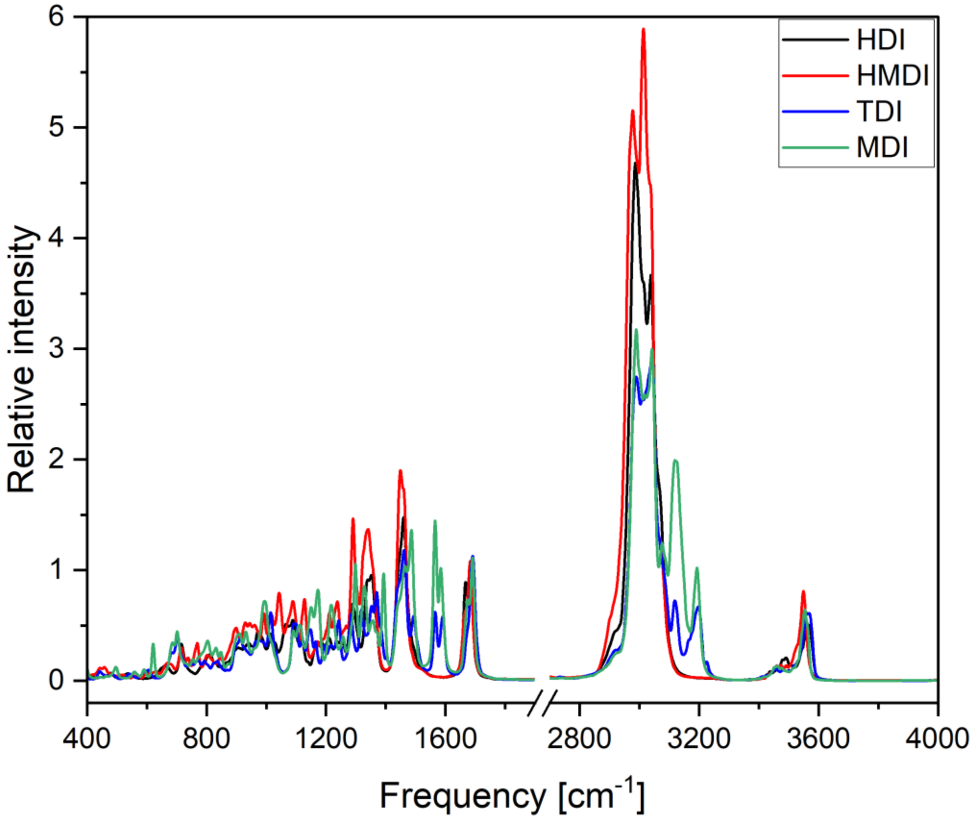

Figure S6. Power spectra of all computational models.

S4. ETS Energy Decomposition Analysis for the TDI and the MDI model

TDI

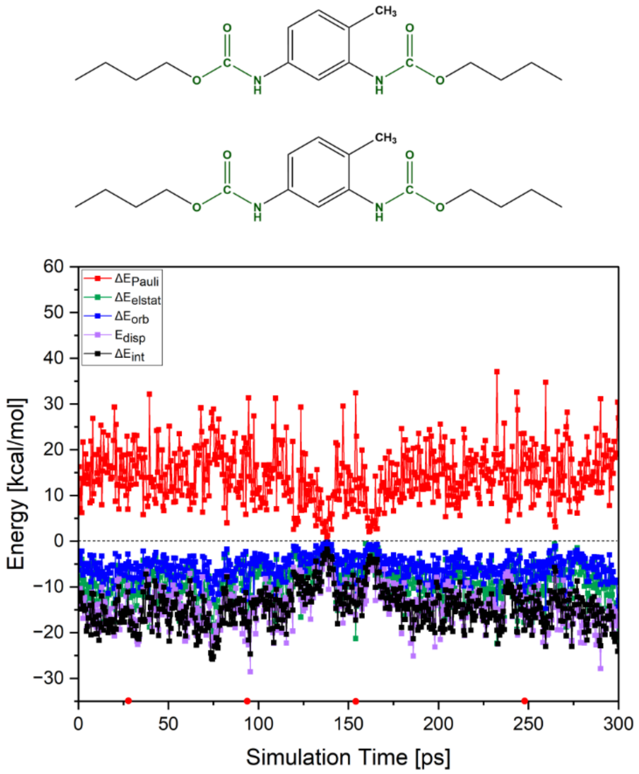

Figure S7. Energy decomposition analysis of snapshots from MD simulations of the TDI model. Small red dots on the X axis depict snapshots for which the differential density and molecular electrostatic potential are demonstrated in **Figure S11**.

MDI

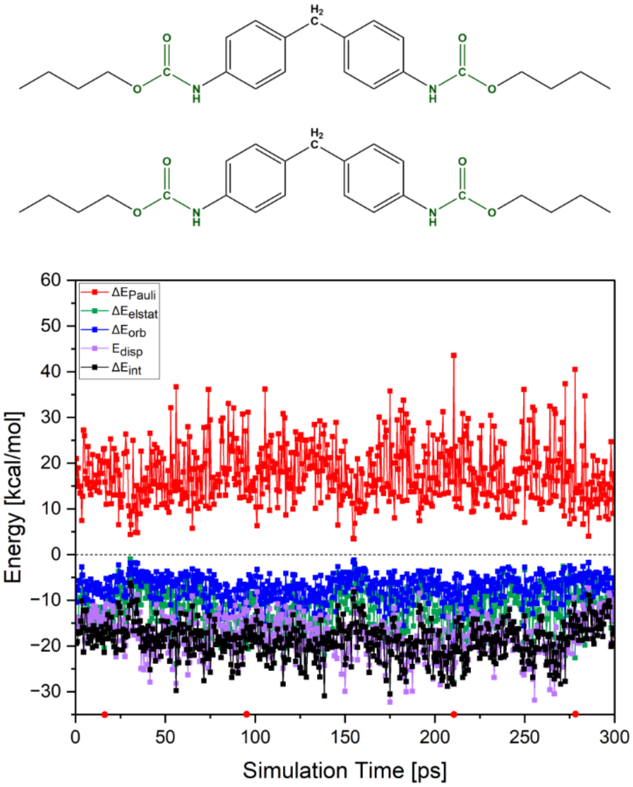

Figure S8. Energy decomposition analysis of snapshots from MD simulations of the MDI model. Small red dots on the X axis depict snapshots for which the differential density and molecular electrostatic potential are demonstrated in **Figure S12**.

S5. Calculation of average values based on the Boltzmann distribution

Average values of interaction energies' components (from the ETS energy decomposition analysis), stabilization energies and their contributions were calculated from corresponding values for snapshots of the simulations every 500 fs. As the total duration of each simulation was 300 ps, every model was analyzed through the 600 snapshots. Quantity values of the selected snapshot were scaled through the probability based on the Boltzmann distribution:

$$X = \sum_i x_i \cdot \frac{e^{-\frac{E_i}{kT}}}{\sum_i e^{-\frac{E_i}{kT}}}$$

where X is the average value of the analyzed quantity, i is the number of snapshots for the total simulation (in this study, i=600), x_i is the quantity value for the snapshot i, E_i is the total bonding energy of the snapshot i, k is the Boltzmann constant, and T is the absolute temperature of the simulation (in this study, T=300 K).

Table S1. Decomposition of interaction energies between fragments of models (**Figure 3**) from equilibrium state calculations and from simulation snapshots, in kcal/mol.

Model structure	Calculations type	ΔE_{Pauli}	ΔE_{elstat}	ΔE_{orb}	E_{disp}	$\mathbf{\Delta E_{int}}$	$\Delta E_{int} - E_{disp}$
**HDI**	Equilibrium state	26.62	−15.79	−8.70	−22.23	**−20.10**	2.13
	MD	17.57	−12.22	−7.36	−15.17	**−17.18**	−2.01
**HMDI**	Equilibrium state	33.67	−13.36	−9.46	−31.49	**−20.64**	10.85
	MD	16.62	−9.87	−6.51	−15.51	**−15.27**	0.24
**TDI**	Equilibrium state	39.16	−19.75	−11.14	−35.60	**−27.33**	8.27
	MD	13.85	−8.61	−5.50	−13.79	**−14.05**	−0.26
**MDI**	Equilibrium state	44.72	−18.96	−12.03	−44.80	**−31.07**	13.73
	MD	17.13	−11.12	−6.84	−17.29	**−18.12**	−0.83

S6. Stabilization energy contributions for the TDI and the MDI model

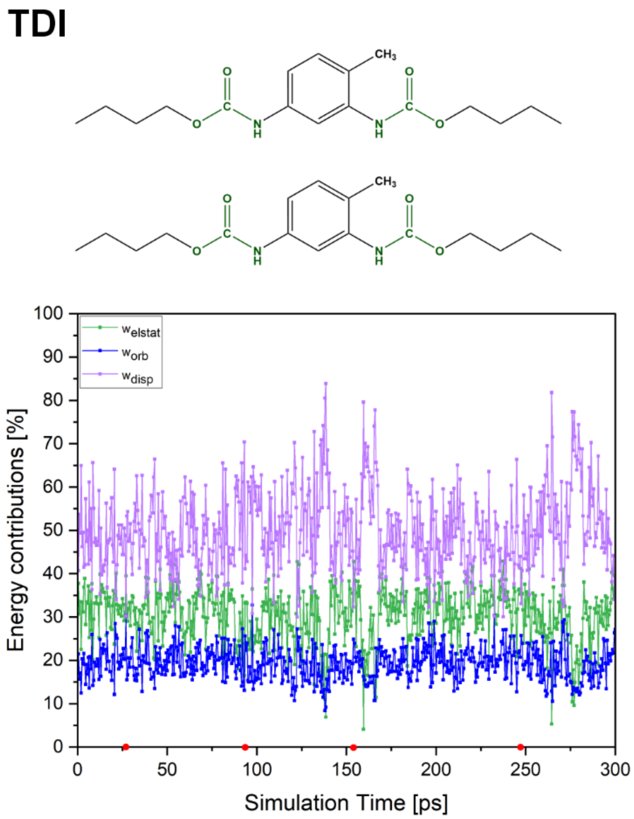

Figure S9. Stabilization energy contributions of snapshots from MD simulations of the TDI model. Small red dots on the X axis depict snapshots for which the differential density and molecular electrostatic potential are demonstrated in **Figure S11**.

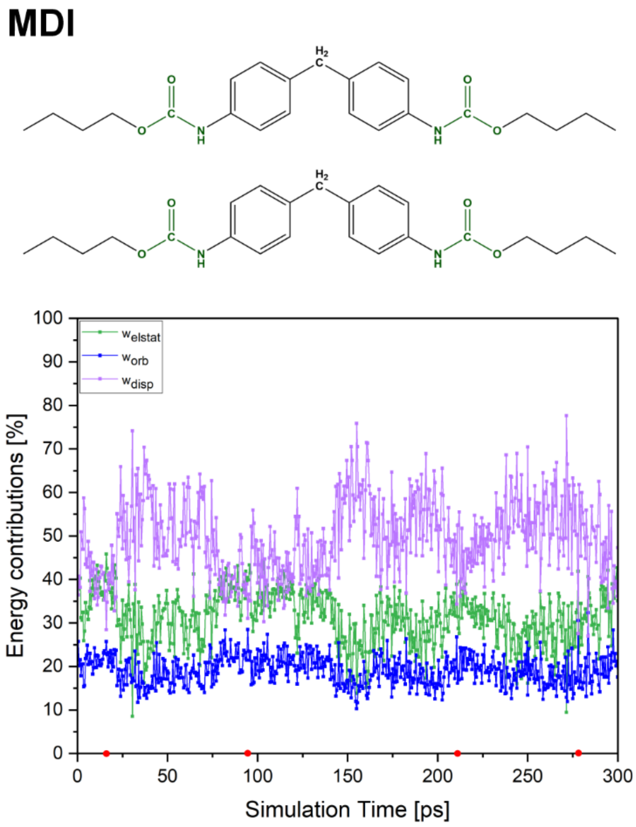

Figure S10. Stabilization energy contributions of snapshots from MD simulations of the MDI model. Small red dots on the X axis depict snapshots for which the differential density and molecular electrostatic potential are demonstrated in **Figure S12**.

Table S2. Comparison of average stabilization energies ΔE_{stab} (in kcal/mol) and their components (in %) between fragments of models (**Figure 3**) from equilibrium state calculations and from simulation snapshots.

Model structure	Calculations type	ΔE_{stab}	w_{elstat}	w_{orb}	w_{disp}
**HDI**	Equilibrium state	−46.72	33.8	18.6	47.6
	MD	−34.74	34.6	20.6	44.8
**HMDI**	Equilibrium state	−54.31	24.6	17.4	58.0
	MD	−31.90	29.7	19.8	50.5
**TDI**	Equilibrium state	−66.49	29.7	16.8	53.5
	MD	−27.89	29.7	19.0	51.3
**MDI**	Equilibrium state	−75.79	25.0	15.9	59.1
	MD	−35.25	31.2	19.0	49.8

S7. Differential densities and molecular electrostatic maps for the TDI and the MDI model

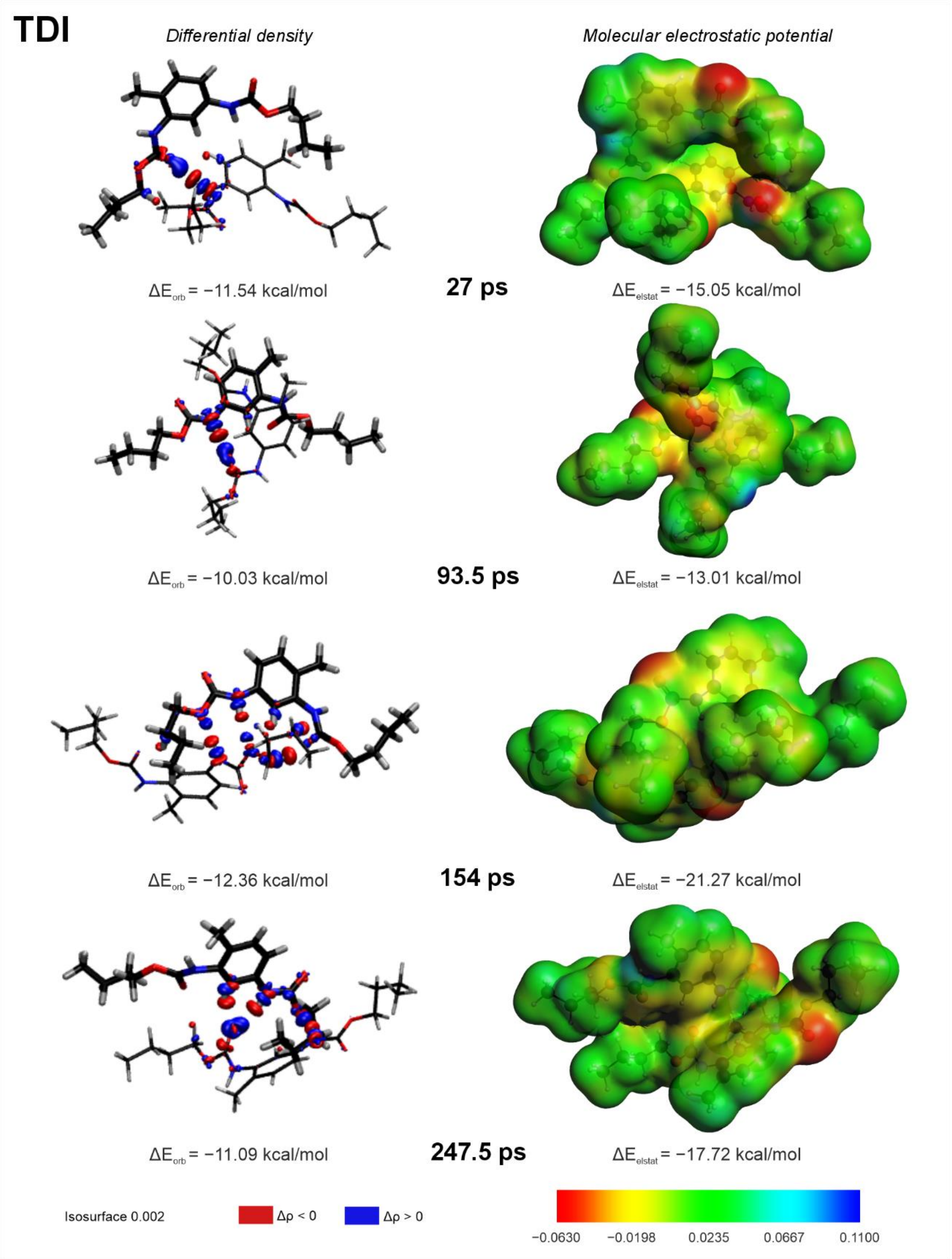

Figure S11. Differential densities (left panel) and molecular electrostatic potential maps (right panel) of chosen snapshots from the MD simulation of the TDI model; different thicknesses of lines are used only to distinguish individual model's chains. The isosurface value is set to 0.002.

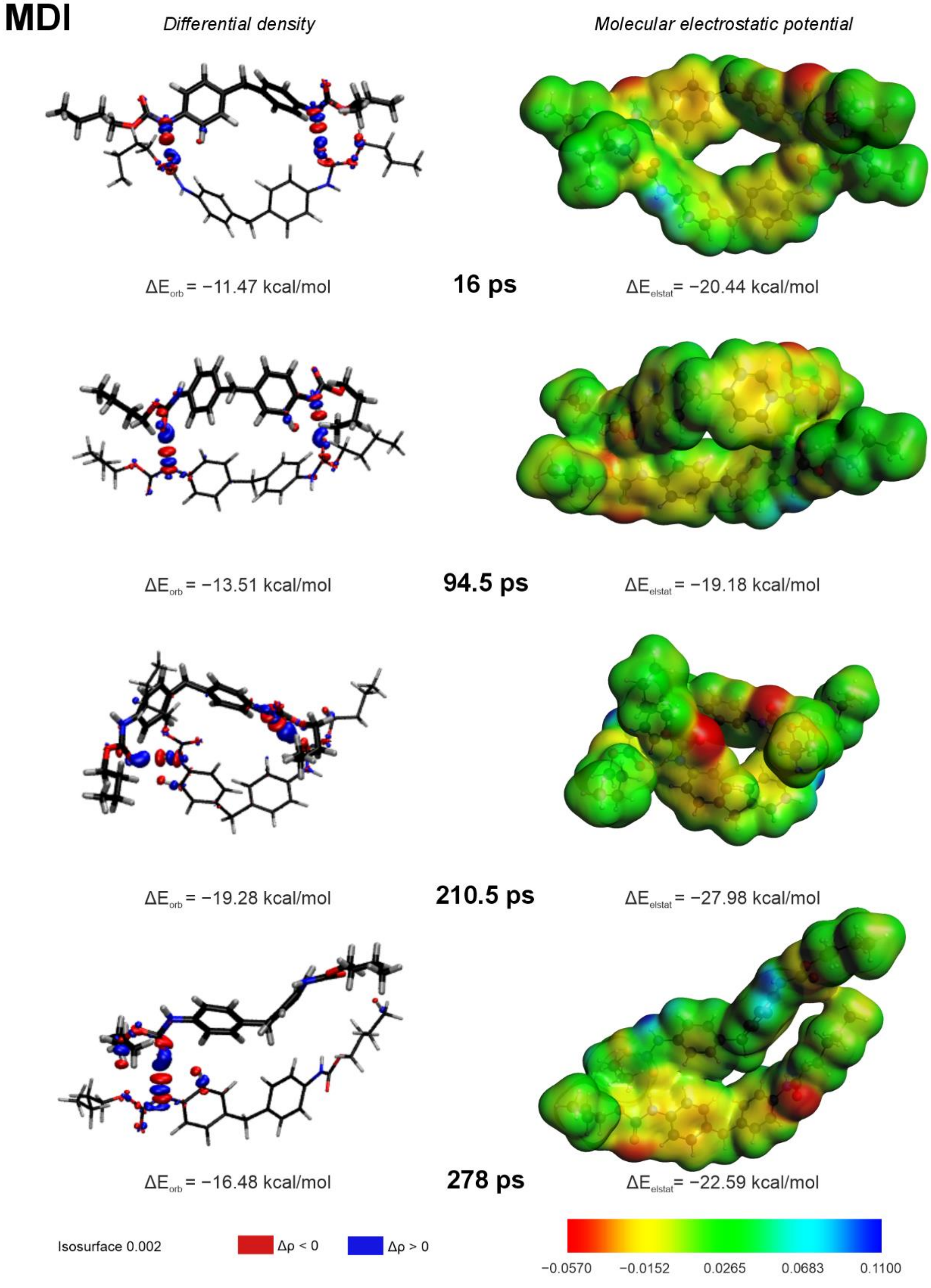

Figure S12. Differential densities (left panel) and molecular electrostatic potential maps (right panel) of chosen snapshots from the MD simulation of the MDI model; different thicknesses of lines are used only to distinguish individual model's chains. The isosurface value is set to 0.002.

S8. Noncovalent Interactions calculations

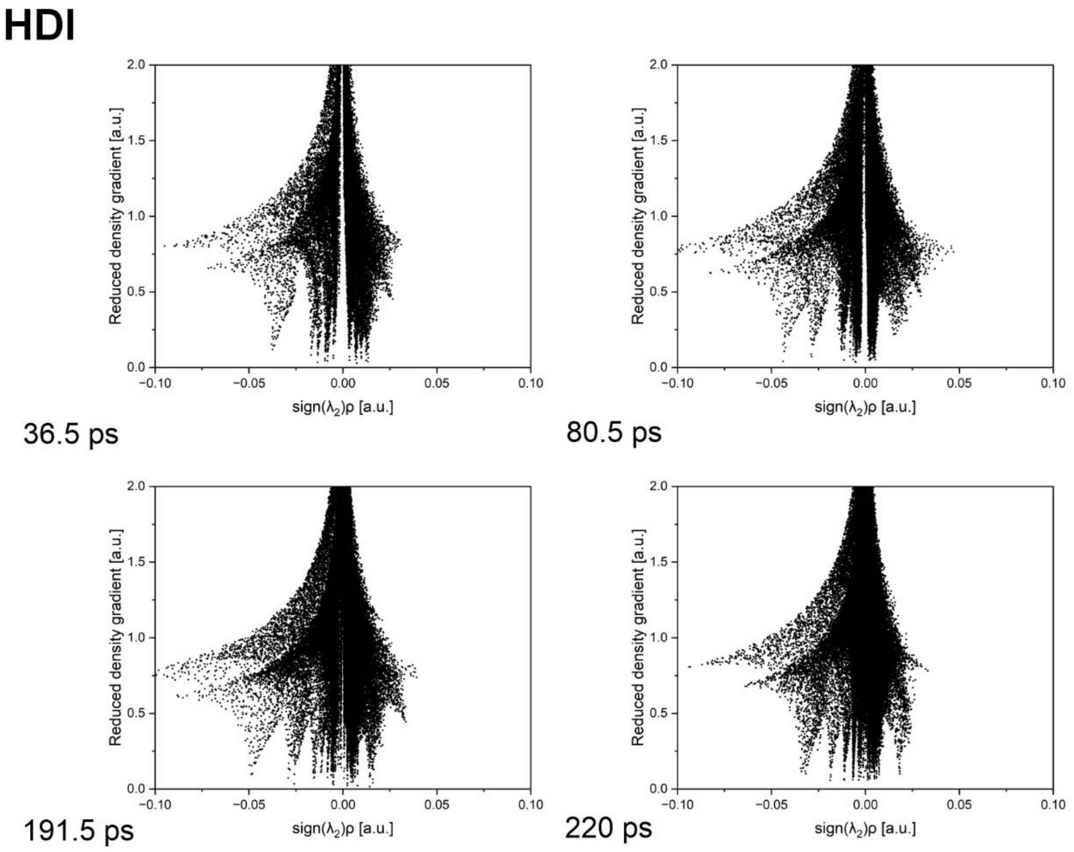

Figure S13. Reduced density gradient of the HDI model snapshots versus electron density multiplied by the sign of the second eigenvalue λ_2 of the Hessian.

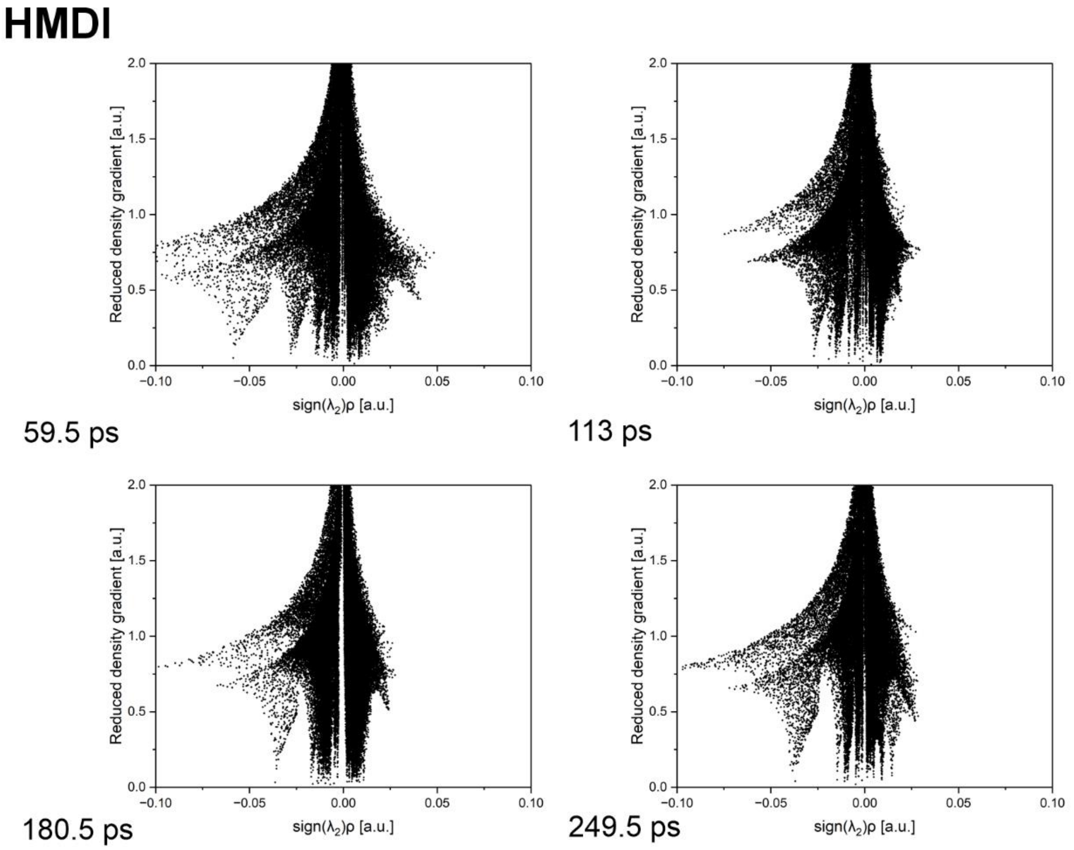

Figure S14. Reduced density gradient of the HMDI model snapshots versus electron density multiplied by the sign of the second eigenvalue λ_2 of the Hessian.

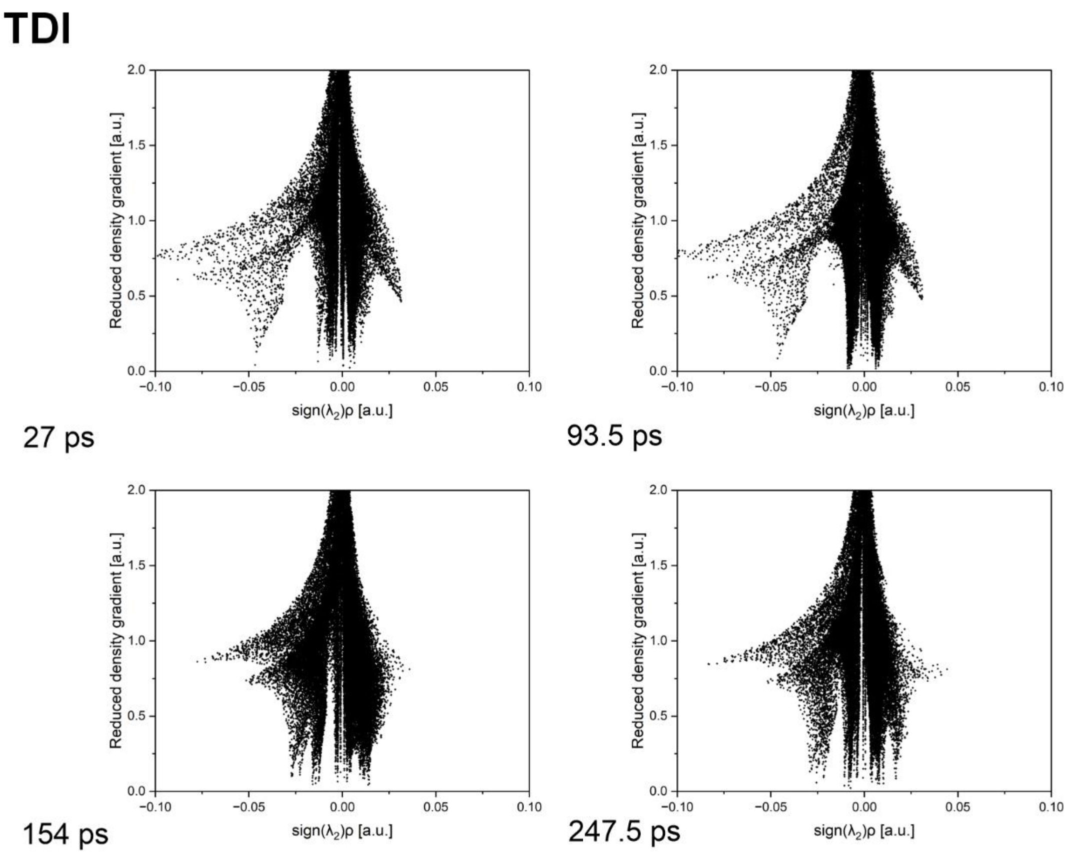

Figure S15. Reduced density gradient of the TDI model snapshots versus electron density multiplied by the sign of the second eigenvalue λ_2 of the Hessian.

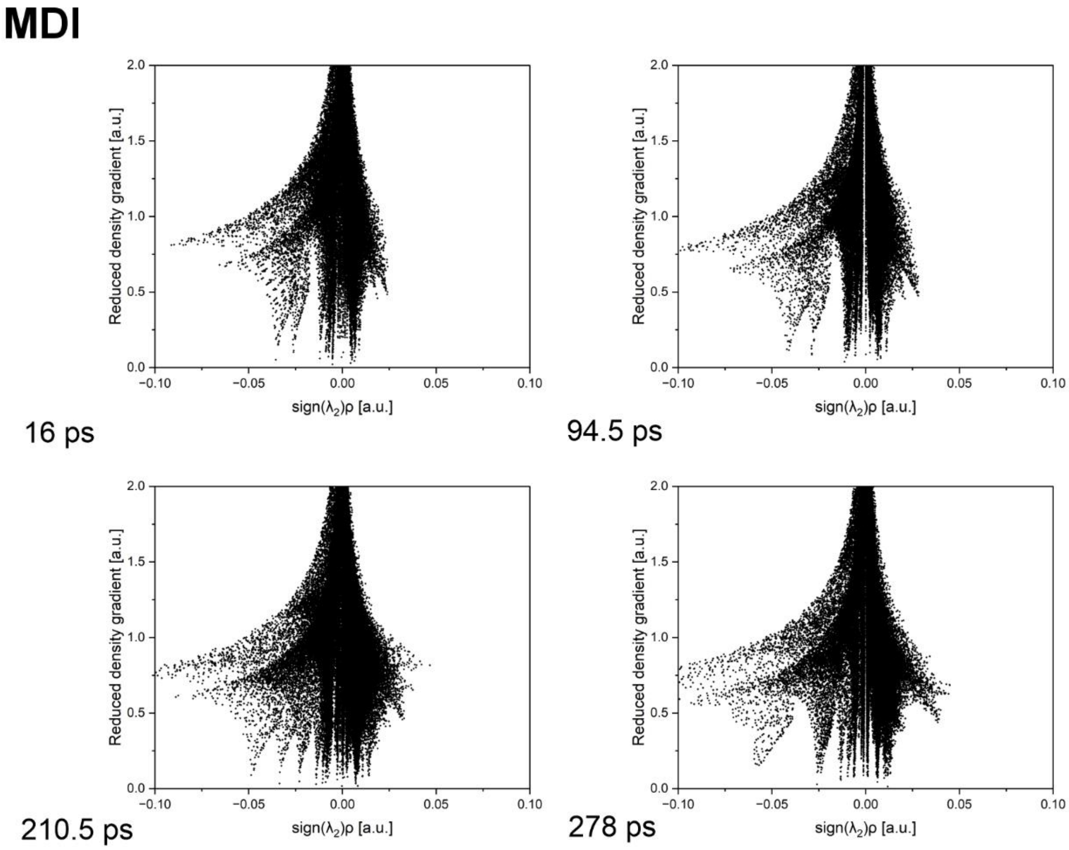

Figure S16. Reduced density gradient of the MDI model snapshots versus electron density multiplied by the sign of the second eigenvalue λ_2 of the Hessian.

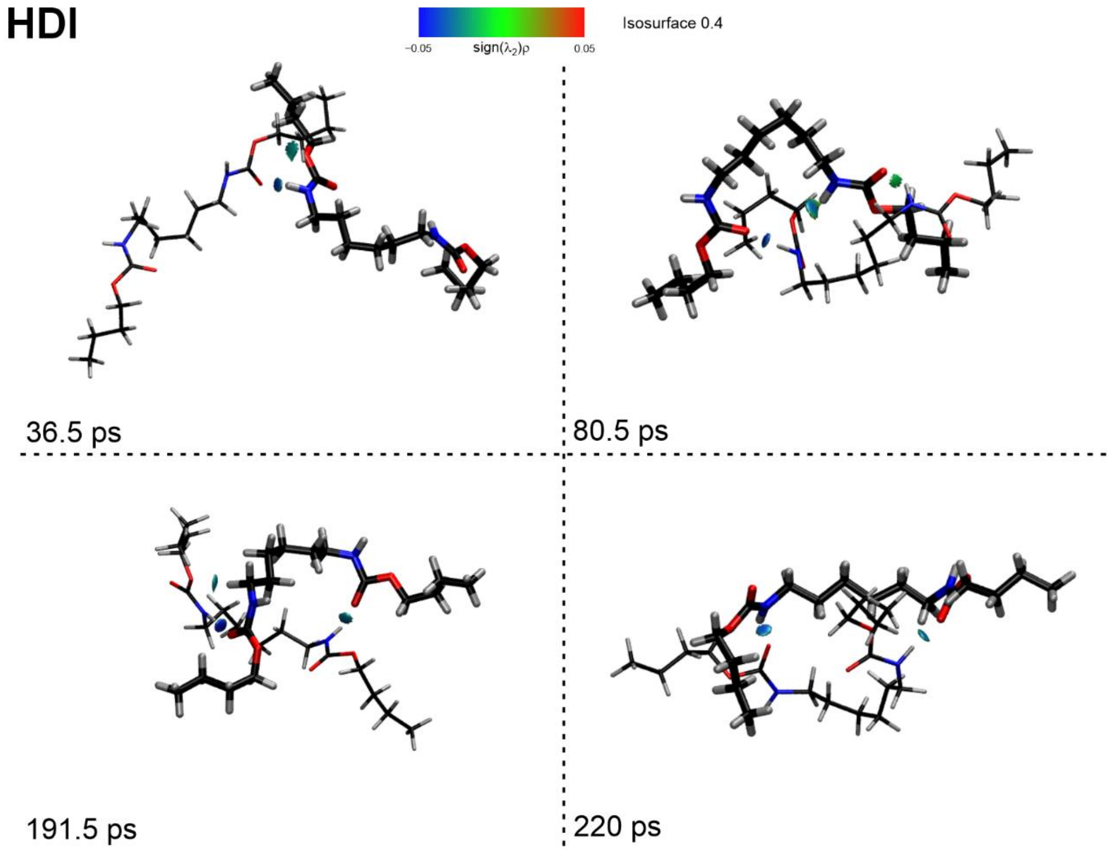

Figure S17. Density gradient isosurfaces for the HDI model snapshots. The BGR color scheme is used for the values of sign(λ_2)ρ ranging from -0.05 to 0.05. Blue color depicts strong attractive interactions, while green color depicts weak attractive interactions. Dispersion interactions between the model chains are neglected for clarity.

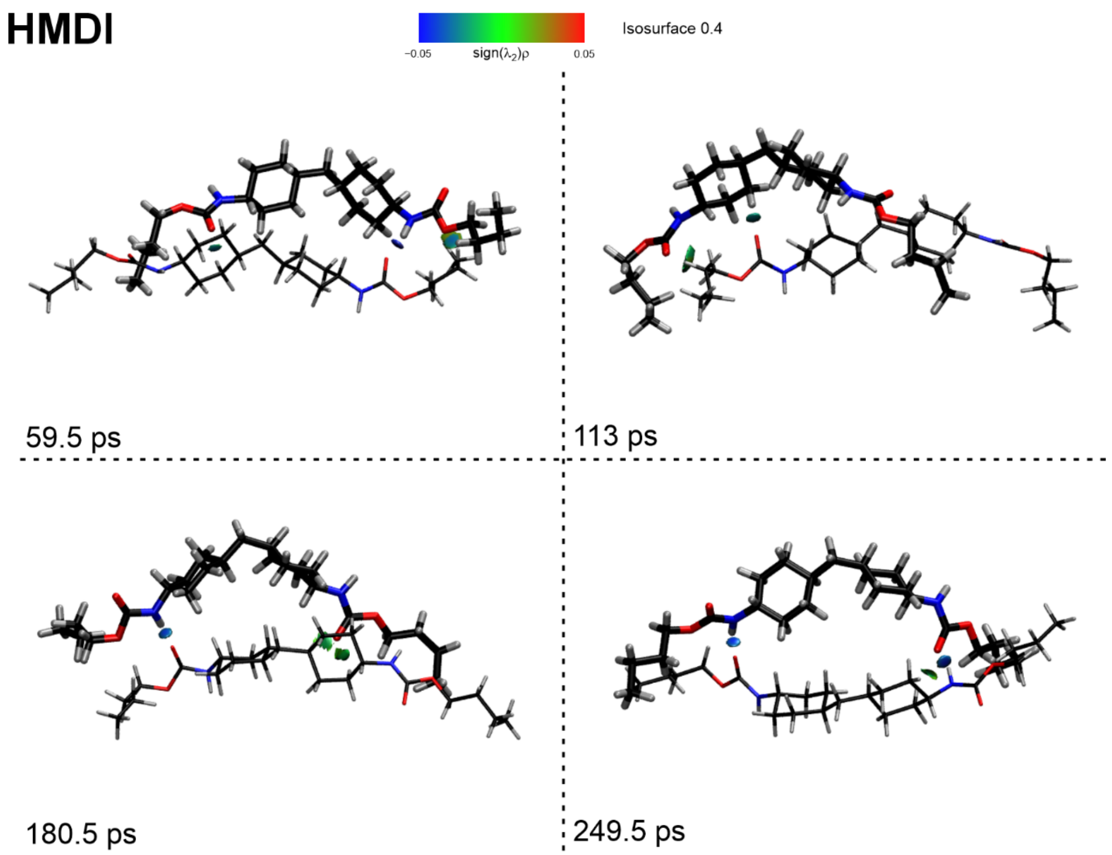

Figure S18. Density gradient isosurfaces for the HMDI model snapshots. The BGR color scheme is used for the values of sign(λ_2)ρ ranging from -0.05 to 0.05. Blue color depicts strong attractive interactions, while green color depicts weak attractive interactions. Dispersion interactions between the model chains are neglected for clarity.

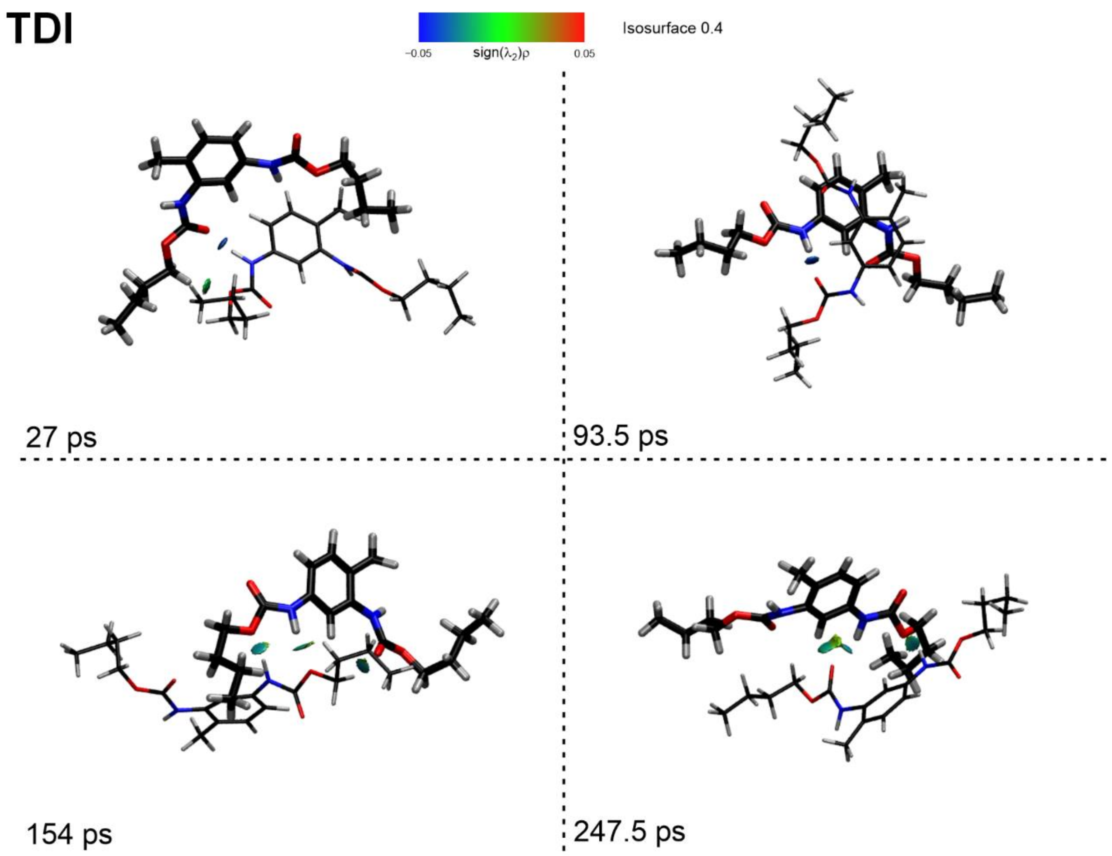

Figure S19. Density gradient isosurfaces for the TDI model snapshots. The BGR color scheme is used for the values of sign(λ_2)ρ ranging from -0.05 to 0.05. Blue color depicts strong attractive interactions, while green color depicts weak attractive interactions. Dispersion interactions between the model chains are neglected for clarity.

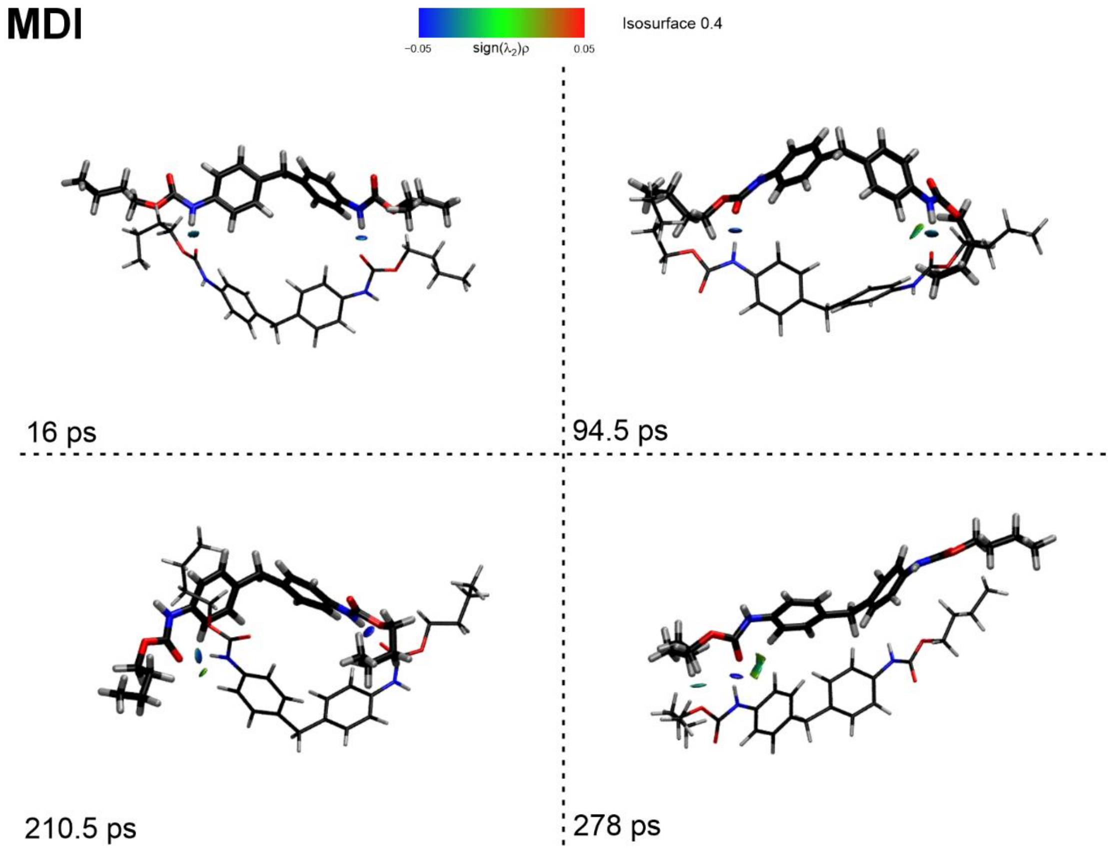

Figure S20. Density gradient isosurfaces for the MDI model snapshots. The BGR color scheme is used for the values of sign(λ_2)ρ ranging from -0.05 to 0.05. Blue color depicts strong attractive interactions, while green color depicts weak attractive interactions. Dispersion interactions between the model chains are neglected for clarity.

S9. Number and strength of HBs during MD simulations

Table S3. Number and strength of HBs during MD simulations of computational models.

Model structure	Simulation time [ps]	Interaction energy for the snapshot [kcal/mol]	Number of HBs	Interaction energy per one HB [kcal/mol]
**HDI**	36.5	−10.98	1	−10.98
	80.5	−25.40	3	−8.47
	191.5	−22.47	3	−7.49
	220.0	−21.58	2	−10.79
**HMDI**	59.5	−18.87	3	−6.29
	113.0	−14.16	1	−14.16
	180.5	−23.89	1	−23.89
	249.5	−18.23	2	−9.12
**TDI**	27.0	−15.12	1	−15.12
	93.5	−17.61	1	−17.61
	154.0	−17.24	3	−5.75
	247.5	−19.48	3	−6.49
**MDI**	16.0	−21.37	2	−10.69
	94.5	−20.37	2	−10.19
	210.5	−28.34	2	−14.17
	278.0	−13.38	1	−13.38

Average values of interaction energy per one hydrogen bond were calculated as the arithmetic mean of corresponding energies for the four analyzed snapshots. The order of average values is as follows: HMDI (−13.4 kcal/mol) < MDI (−12.1 kcal/mol) < TDI (−11.2 kcal/mol) < HDI (−9.4 kcal/mol).
